# Supplementary material for: Solid tumor immunotherapy using NKG2D-based adaptor CAR T cells
Source: Cell Rep Med. 2024 Nov 19;5(11):101827. doi: 10.1016/j.xcrm.2024.101827 (PMC11604534; doi:10.1016/j.xcrm.2024.101827)
Supplement: Document S1. Figures S1–S7 [file mmc1.pdf]

Cell Reports Medicine, Volume *5*

Supplemental information

Solid tumor immunotherapy

using NKG2D-based adaptor CAR T cells

Jana Obajdin, Daniel Larcombe-Young, Maya Glover, Fahima Kausar, Caroline M. Hull, Katie R. Flaherty, Ge Tan, Richard E. Beatson, Phoebe Dunbar, Roberta Mazza, Camilla Bove, Chelsea Taylor, Andrea Bille, Katelyn M. Spillane, Domenico Cozzetto, Alessandra Vigilante, Anna Schurich, David M. Davies, and John Maher

Supplemental Information

Solid tumor immunotherapy using NKG2D-based adaptor CAR

T-cells

Jana Obajdin, Daniel Larcombe-Young, Maya Glover, Fahima Kausar, Caroline M. Hull, Katie R. Flaherty, Ge Tan, Richard E. Beatson,, Phoebe Dunbar, Roberta Mazza, Camilla Bove, Chelsea Taylor, Andrea Bille, Katelyn M. Spillane, Domenico Cozzetto, Alessandra Vigilante, Anna Schurich, David M. Davies, John Maher

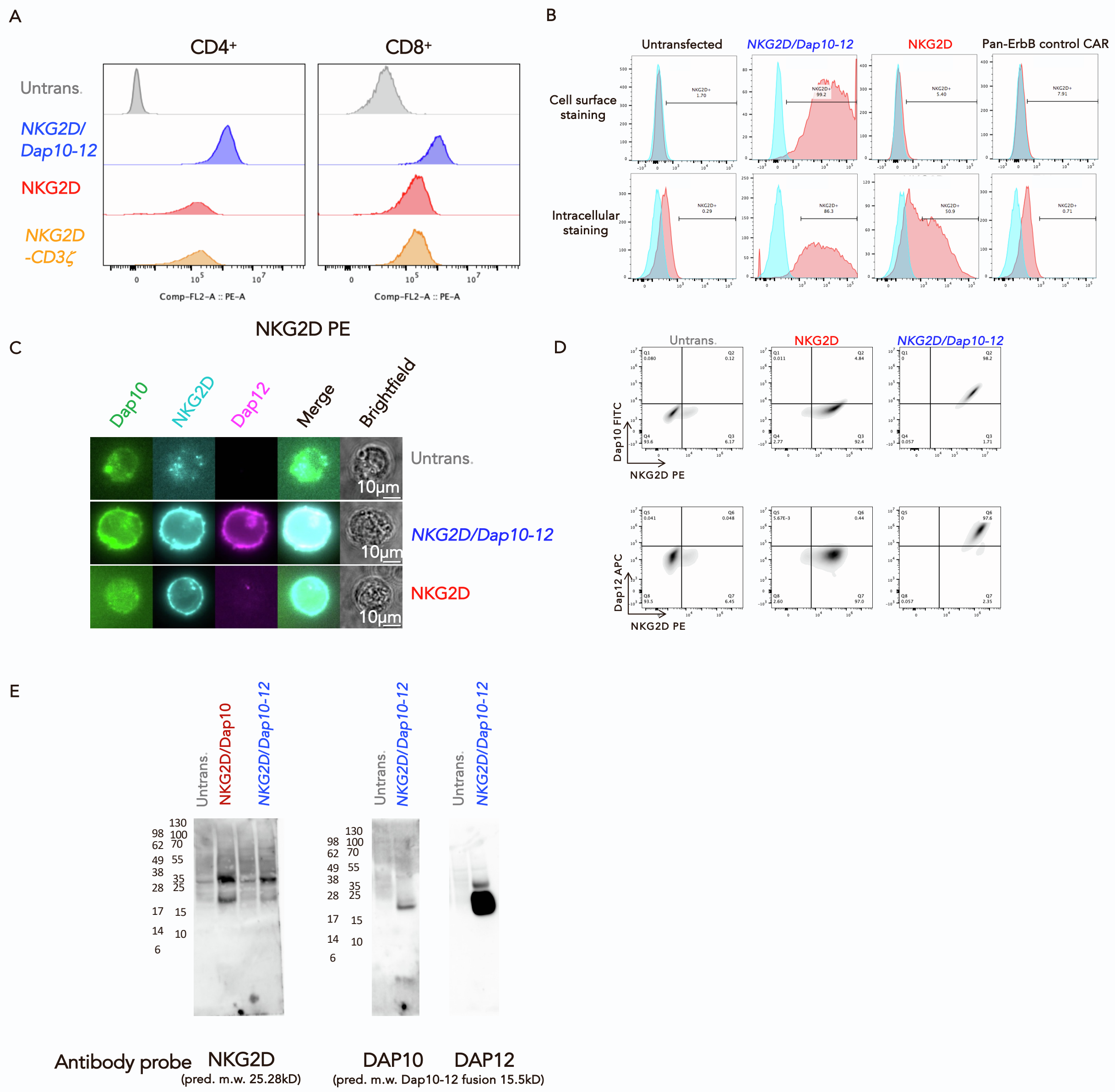

Figure S1. Confirmation of complex formation by NKG2D and Dap10-12 in *NKG2D/Dap10-12* T-cells

(A) Representative flow cytometric analysis of CD4$^+$ and CD8$^+$ T-cells engineered to express the *NKG2D/Dap10-12* CAR or *NKG2D-CD3ζ* CAR. T-cells in which NKG2D alone was over-expressed by retroviral transduction are shown as controls, together with untransduced (untrans.) T-cells. Data are representative of at least 3 independent replicates that gave similar results. (B) 293T cells were transfected with the indicated plasmids. NKG2D expression was detected by flow cytometry in non-permeabilized (cell surface staining) and permeabilized (intracellular staining) cells. A panErbB CAR plasmid (SFG T4) was used as an additional negative control. Data are representative of 3 independent replicates. (C) TIRF microscopy images of the indicated T-cell populations following incubation with Alexa Fluor® 488 anti-human DAP10 antibody, Alexa Fluor® 647 anti-human DAP12 antibody and PE anti-human NKG2D antibodies. Data are representative of 3 independent donors. (D) Upper panels show flow cytometric detection of NKG2D and Dap10 co-expression on the cell surface of the indicated non-permeabilized T-cell populations. Lower panels show co-expression of NKG2D and Dap12 in permeabilized *NKG2D/Dap10-12* T-cells. Data are representative of 3 independent replicates that gave similar results. (E) Western blotting was performed on lysates prepared from 293T cells that were untransfected (untrans.) or transfected with NKG2D/ Dap10-12. NKG2D/Dap10 is a control in which NKG2D is co-expressed with Dap10 alone. Predicted molecular weights of NKG2D and Dap10-12 without post translational modification are provided together with two molecular weight ladders run along with each blot.

Related to Figure 1.

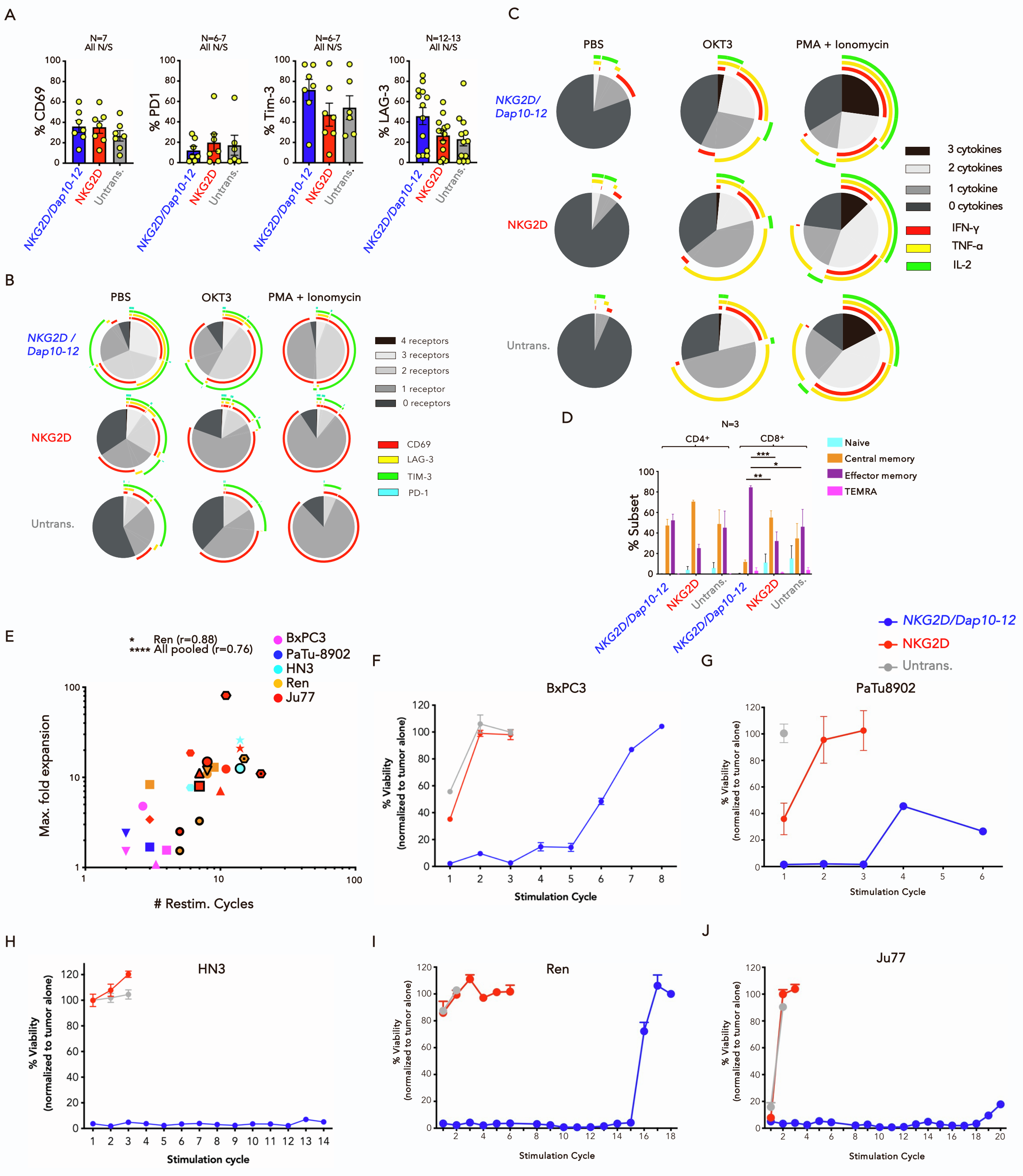

Figure S2. In vitro analysis of *NKG2D/Dap10-12* T-cells

(A) Flow cytometric analysis of CD69, PD1, Tim-3 and LAG-3 (all show mean +̲SEM) expression on the indicated CAR and control T-cells. Number of independent biological replicates are indicated on this and subsequent panels as appropriate. N/S – not significant. (B) Spice analysis of activation/ exhaustion marker expression by T-cells engineered to express *NKG2D/Dap10-12*, NKG2D alone or untransduced as control. Plots are representative of two independent repeats. (C) *NKG2D/Dap10-12* CAR T-cells were stimulated for 24 hours as indicated, making comparison with the indicated control cells. Cytokine-producing cells were quantified using flow cytometry. Data are representative of three independent replicates. (D) Differentiation subset analysis of *NKG2D/Dap10-12* CAR T-cells on day 12 of culture, making comparison with the indicated control cells (mean +̲ SEM). $CD4^+$ and $CD8^+$ T-cell subsets were defined as naive ($CD45RO^-$ $CCR7^+$), central memory (CM; $CD45RO^+$ $CCR7^+$), effector memory (EM; $CD45RO^+$ $CCR7^-$) and terminally differentiated effector memory (TEMRA) cells ($CD45RO^-$ $CCR7^-$). *$p<0.05$; **$p<0.01$; ***$p<0.001$ by two-way ANOVA. (E) Correlation was assessed between number of productive re-stimulation cycles for individual donors (denoted by different symbols) and maximum fold expansion of *NKG2D/Dap10-12* CAR T-cells on the indicated tumor monolayers (denoted by indicated colors). *$p<0.05$; ****$p<0.0001$ by Spearman test for each individual tumor cell line and for a pooled analysis for all five tumor cell monolayers. Representative tumor re-stimulation assays performed on BxPC3 (F), PaTu8902 (G), HN3 (H), Ren (I) and Ju77 (J) tumor cell monolayers.

Related to Figure 1.

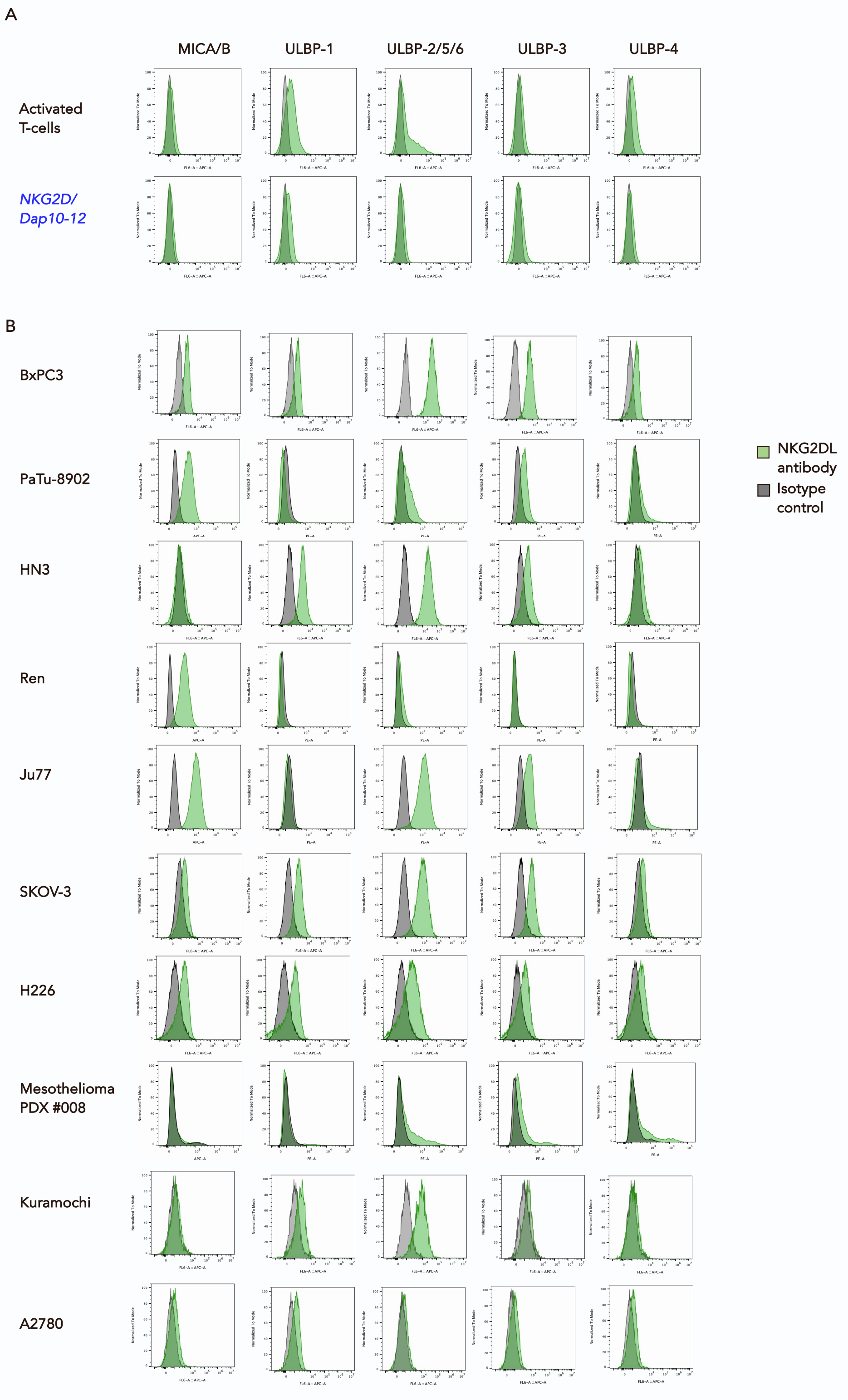

Figure S3. NKG2D ligand expression on tumor cell models and activated T-cells

Activated untransduced or *NKG2D/Dap10-12* CAR T-cells (A) and tumor cells used for in vitro and in vivo studies (B) were analyzed for expression of NKG2D ligands by flow cytometry. Data are representative of at least three independent replicates.

Related to Figure 1.

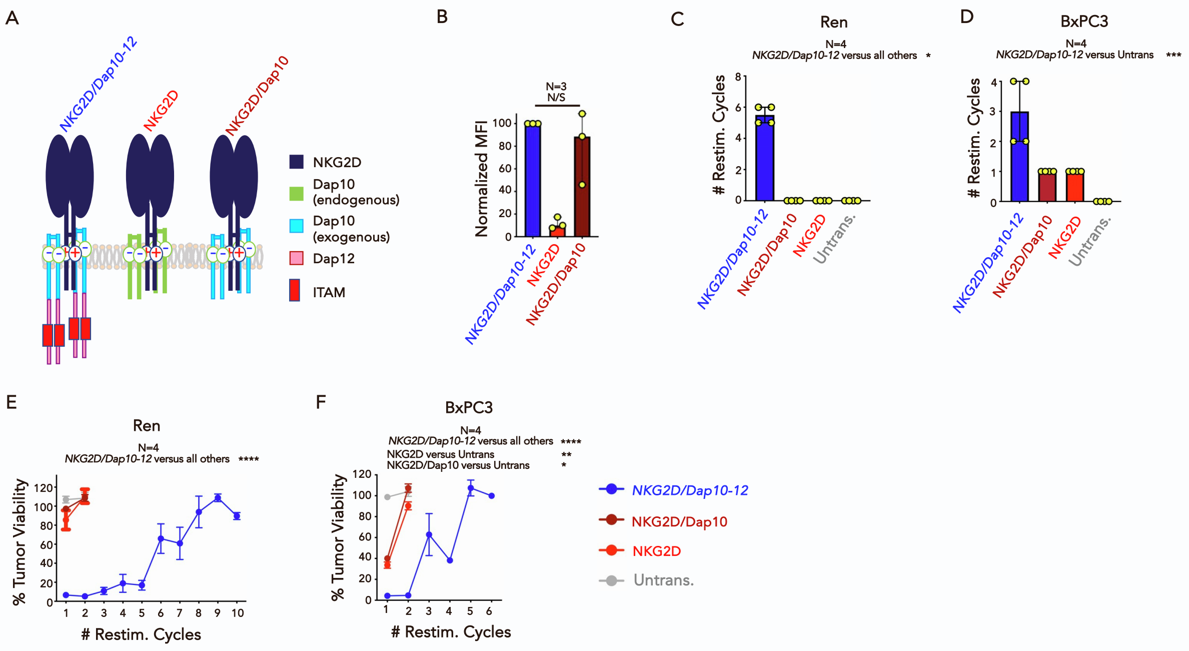

Figure S4. Comparison of *NKG2D/Dap10-12* with NKG2D/Dap10 control T-cells

(A) Function of *NKG2D/Dap10-12* CAR T-cells was compared to controls in which NKG2D alone or the combination of NKG2D and Dap10 were over-expressed. Note that NKG2D also associates with endogenous Dap10 in T-cells. (B) MFI of NKG2D expression in CD4$^+$ T-cells following transduction with the *NKG2D/Dap10-12* CAR, NKG2D alone or NKG2D/Dap10. Number of independent biological replicates is shown on this and subsequent panels as appropriate. Data were normalized to expression in *NKG2D/Dap10-12* CAR T-cells, which was set to 100. Error bars show median + interquartile range. Statistical analysis was by Kruskal-Wallis test; N/S – not significant. Number of effective re-stimulation (restim.) cycles achieved by *NKG2D/Dap10-12* and control T-cells when iteratively re-stimulated twice weekly on Ren (C) and BxPC3 (D) tumor cell lines. Re-stims were considered successful if <60% of tumor cells remained viable at the time of initiation of the next twice weekly stimulation cycle. Data show median + interquartile range. *p<0.05; ***p<0.001 by Kruskal Wallis test. Tumor viability was monitored after each restim cycle on Ren (E) and BxPC3 (F). Data show mean±SEM. *p<0.05; **p<0.01; ***p<0.001 by two-way ANOVA.

Related to Figure 1.

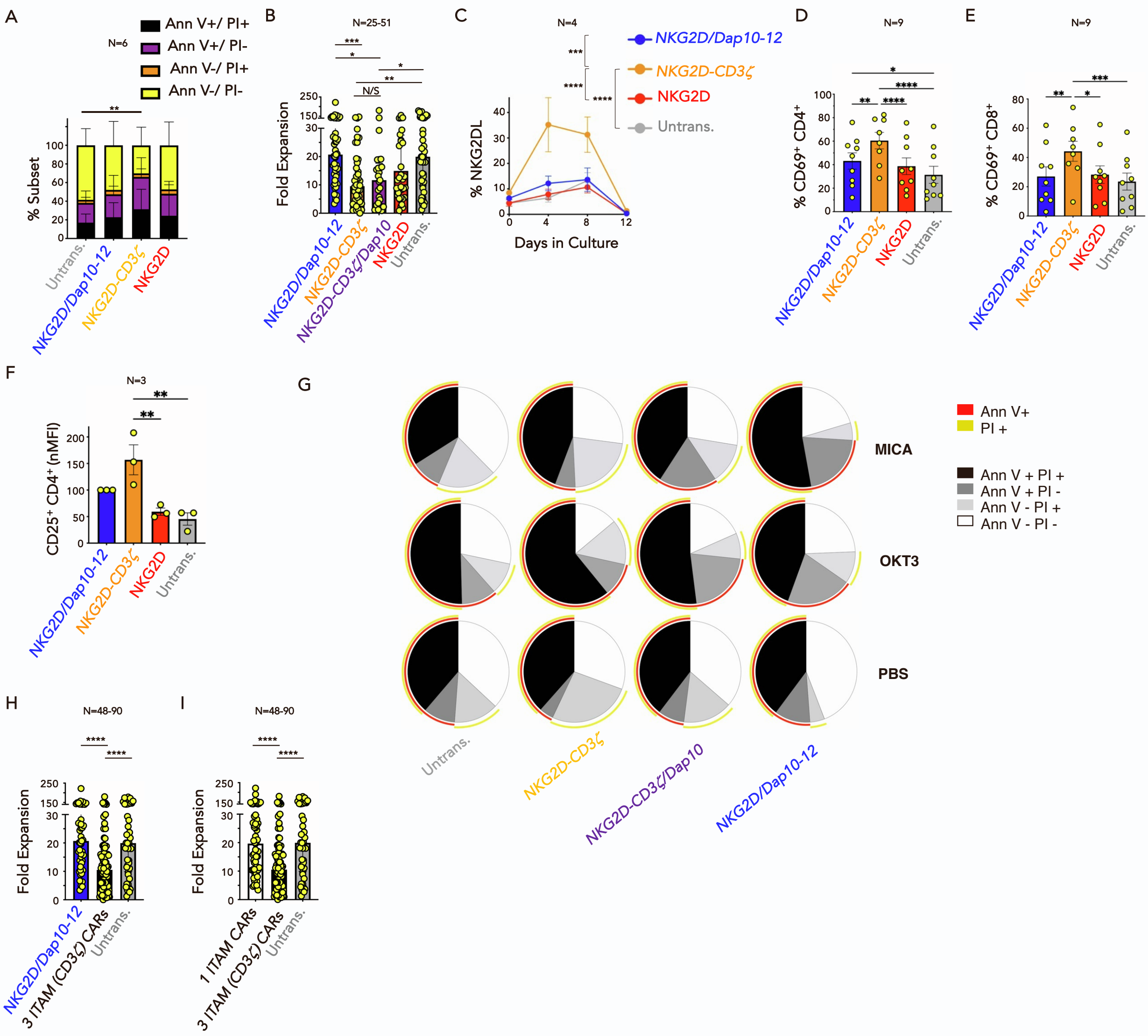

Figure S5. Analysis of viability, yield and activation state of the indicated CAR T-cell populations

(A) T-cells were engineered to express the indicated CARs or NKG2D alone and were analyzed for viability/ apoptosis by flow cytometry following staining with Annexin V (Ann V) and propidium iodide (PI). Mean +/- SD n=6 independent biological replicates. **$p<0.01$ using paired Student t-test for viable (i.e. Ann V-/ PI-) cells. (B) Expansion of the indicated CAR T-cell populations over 11 days post retroviral transduction. Number of independent biological replicates is shown on this and subsequent panels as appropriate. *$p<0.05$, **$p<0.01$, ***$p<0.001$ and N/S (not significant) by Kruskal-Wallis test. (C) Engineered T-cells were expanded in culture and analyzed for NKG2DL expression by flow cytometry using an NKG2D-Fc fusion protein to simultaneously detect all ligands (mean $\pm$ SEM; ***$p<0.001$, ****$p<0.001$ by two-way ANOVA. Following expansion in culture, the indicated CAR T-cells were analyzed by flow cytometry for CD69 (CD4$^+$ T-cells, D; CD8$^+$ T-cells, E) and CD25 (CD4$^+$ T-cells, F). In the case of CD25, since all cells were positive, MFI was normalized against that of *NKG2D/Dap10-12* which was set to 100 arbitrary units. *$p<0.05$, **$p<0.01$, ***$p<0.001$ and ****$p<0.0001$ by one-way ANOVA. (G) T-cells that expressed the indicated CARs or NKG2D as control were stimulated for 24 hours on immobilized MICA, OKT3 or with PBS as control. To quantify activation-induced cell death, cells were analyzed for viability/ apoptosis by flow cytometry following staining with Ann V and PI. (H) Expansion of the indicated CAR T-cell populations over 11 days post retroviral transduction. The 3 ITAM (CD3ζ) CARs group comprised *NKG2D-CD3ζ*, *NKG2D-CD3ζ/Dap10* and *NKG2D/Dap10-CD3ζ*. ****$p<0.0001$ by Kruskal-Wallis test. (I) Expansion of the indicated CAR T-cell populations over 11 days post retroviral transduction. The 1 ITAM CARs group comprised *NKG2D/Dap10-12*, *NKG2D-Dap10-12*, *N(Tr.)-Dap10-12*, *NKG2D-Dap10-12/Dap10*, *N(Tr.)-Dap10-12/Dap10* and *NKG2D/Dap10-CD3ζ(1XX)*. The 3 ITAM (CD3ζ) CARs group comprised *NKG2D-CD3ζ*, *NKG2D-CD3ζ/Dap10* and *NKG2D/Dap10-CD3ζ*. ****$p<0.0001$ by Kruskal-Wallis test.

Related to Figure 3.

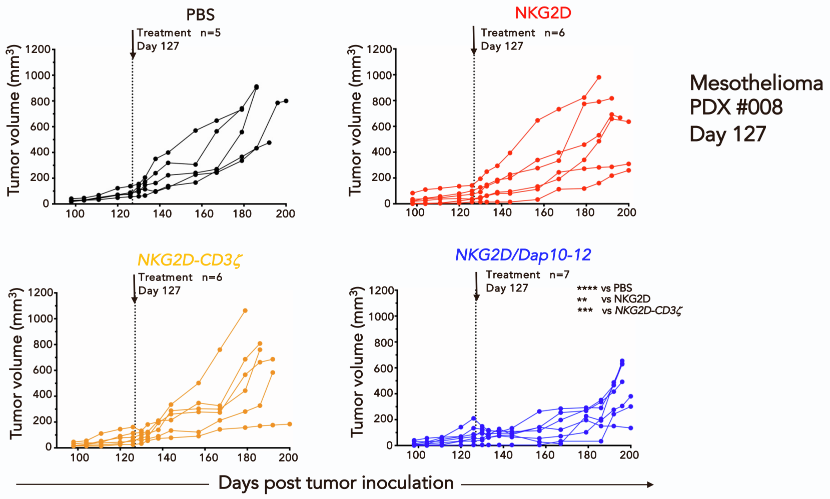

Figure S6. In vivo comparison of *NKG2D/Dap10-12* and *NKG2D-CD3ζ* CAR T-cells against advanced mesothelioma PDX tumors

Small chunks (approximately 2mm x 2mm) of mesothelioma PDX_008 were injected subcutaneously in NSG mice. Once tumors had established for 127 days, 4 million of the indicated CAR T-cell populations were injected i.v., making comparison with PBS and T-cells in which NKG2D alone was over-expressed. Number of independent biological replicates is indicated. Tumor volume was monited by caliper measurements. $^{**}p<0.01$; $^{***}p<0.001$; $^{****}p<0.0001$ using two-way ANOVA.

Related to Figure 4.

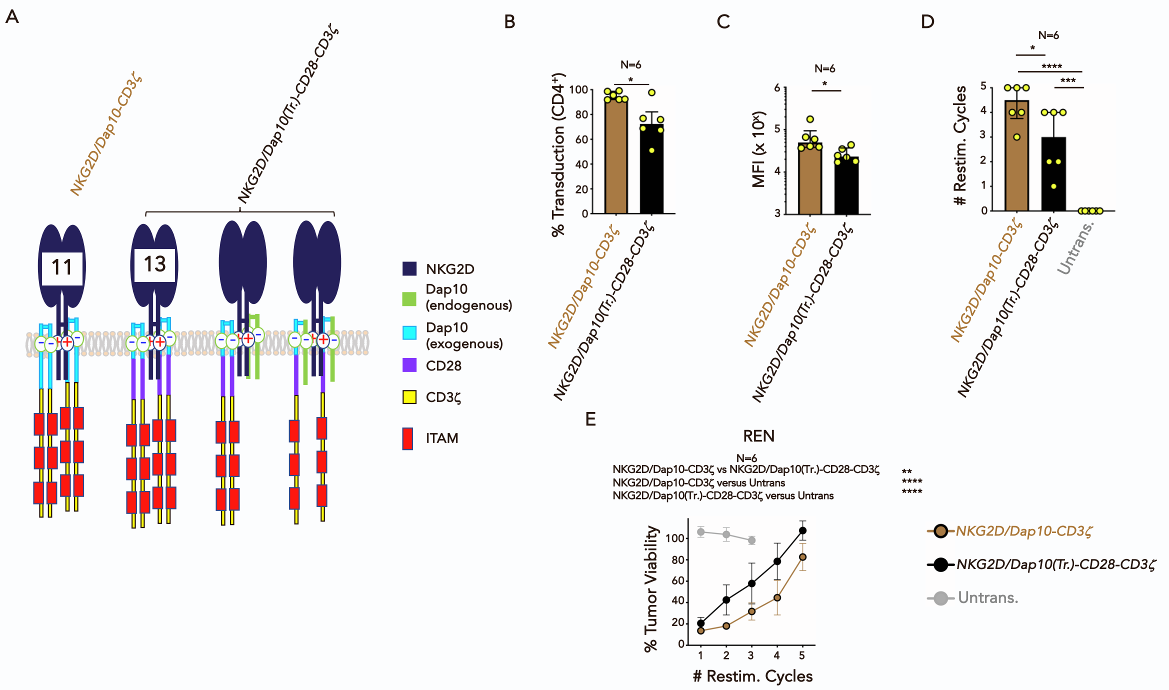

Figure S7. Evaluation of CD28-containing NKG2D-based CAR designs

(A) Function of CD28 versus Dap10 was compared using the indicated CAR designs. Numbering shown is to match these CARs with those shown in Figure 5. Additional predicted structures shown for *NKG2D/Dap10(Tr.)-CD28-CD3ζ* reflect the ability of NKG2D to also associated with endogenous Dap10 present in T-cells. (B) Transduction efficiency of the indicated CARs was assessed by flow cytometry, measuring percentage cell surface NKG2D in the $CD4^+$ T-cell subset. Number of independent biological replicates is shown on this and subsequent panels. Error bars show median + interquartile range. *$p<0.05$ by Mann Whitney test. (C) Mean fluorescence intensity of NKG2D expression in $CD4^+$ T-cells following transduction with the indicated CARs. Error bars show median + interquartile range. *$p<0.05$ by Mann Whitney test. (D) Number of effective re-stim cycles achieved by the indicated CAR T-cells when iteratively re-stimulated twice weekly on Ren tumor cells. Re-stims were considered successful if <60% of tumor cells remained viable. Data show median + interquartile range. *$p<0.05$; ***$p<0.001$; ****$p<0.0001$ by one-way ANOVA. (E) Tumor viability was monitored after each restim cycle on Ren cells. Data show mean±SEM. **$p<0.01$; ****$p<0.0001$ by two-way ANOVA.

Related to Figure 5.
