# Supplementary material for: Solid tumor immunotherapy using NKG2D-based adaptor CAR T cells
Source: Cell Rep Med. 2024 Nov 19;5(11):101827. doi: 10.1016/j.xcrm.2024.101827 (PMC11604534; doi:10.1016/j.xcrm.2024.101827)
Supplement: Document S2. Article plus supplemental information [file mmc2.pdf]

Cell Reports Medicine

Article

Solid tumor immunotherapy using NKG2D-based adaptor CAR T cells

Graphical abstract

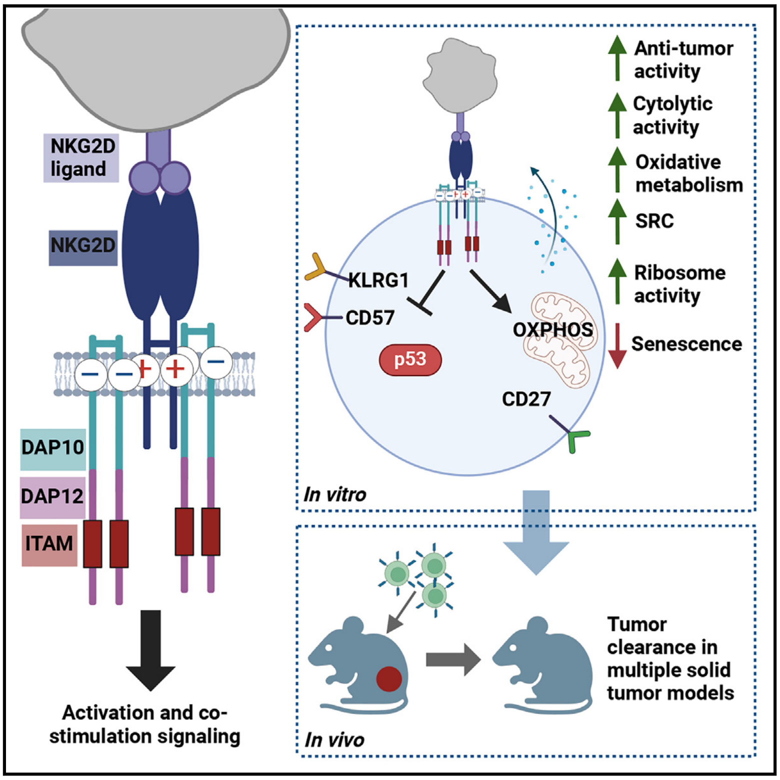

Highlights

- NKG2D naturally associates with the adaptor molecule, Dap10
- Addition of Dap12 to the Dap10 C terminus yields a potent NKG2D-based CAR
- The resulting adaptor CAR configuration promotes functional persistence of T cells
- As a result, adaptor CAR T cells mediate enhanced anti-tumor activity

Authors

Jana Obajdin, Daniel Larcombe-Young, Maya Glover, ..., Anna Schurich, David M. Davies, John Maher

Correspondence

john.maher@kcl.ac.uk

In brief

NKG2D ligands are widely expressed by solid tumors providing an attractive targeting opportunity with NKG2D-based CAR T cells. Here, Obajdin et al. show that this is optimally achieved using an adaptor CAR format in which signaling is provided by highly compact Dap10 and Dap12 signaling units.

Obajdin et al., 2024, Cell Reports Medicine *5*, 101827
November 19, 2024 © 2024 The Author(s). Published by Elsevier Inc.
https://doi.org/10.1016/j.xcrm.2024.101827

CellPress

Cell Reports Medicine

CellPress
OPEN ACCESS

Article

Solid tumor immunotherapy using NKG2D-based adaptor CAR T cells

Jana Obajdin,[1,10] Daniel Larcombe-Young,[1,10] Maya Glover,[2] Fahima Kausar,[2] Caroline M. Hull,[2] Katie R. Flaherty,[3] Ge Tan,[1] Richard E. Beatson,[4] Phoebe Dunbar,[2] Roberta Mazza,[2] Camilla Bove,[2] Chelsea Taylor,[2] Andrea Bille,[5] Katelyn M. Spillane,[6] Domenico Cozzetto,[7] Alessandra Vigilante,[8] Anna Schurich,[3] David M. Davies,[2,10] and John Maher[1,2,9,10,11,*]

[1]King's College London, School of Cancer and Pharmaceutical Sciences, CAR Mechanics Lab, London SE1 9RT, UK
[2]Leucid Bio Ltd, Guy's Hospital, London SE1 9RT, UK
[3]King's College London, Department of Infectious Diseases, School of Immunology and Microbial Sciences, Guy's Hospital, London SE1 9RT, UK
[4]Department of Respiratory Medicine, Division of Medicinal Sciences, University College London, London, UK
[5]Department of Thoracic Surgery, Guy's and St. Thomas' NHS Trust Foundation, London SE1 9RT, UK
[6]Department of Physics, King's College London, London WC2R 2LS, UK
[7]Division of Digestive Diseases, Faculty of Medicine, Imperial College London, London W12 0NN, UK
[8]King's College London, Centre for Stem Cells and Regenerative Medicine & Institute for Liver Studies, Guy's Hospital, London SE1 9RT, UK
[9]Department of Immunology, Eastbourne Hospital, Kings Drive, Eastbourne, East Sussex BN21 2UD, UK
[10]These authors contributed equally
[11]Lead contact
*Correspondence: john.maher@kcl.ac.uk
https://doi.org/10.1016/j.xcrm.2024.101827

SUMMARY

NKG2D ligands (NKG2DLs) are broadly expressed in cancer. To target these, we describe an adaptor chimeric antigen receptor (CAR) termed *NKG2D/Dap10-12*. Herein, T cells are engineered to co-express NKG2D with a fusion protein that comprises Dap10 joined to a Dap12 endodomain. *NKG2D/Dap10-12* T cells elicit compelling efficacy, eradicating or controlling NKG2DL-expressing tumors in several established xenograft models. Importantly, durable responses, long-term survival, and rejection of tumor re-challenge are reproducibly achieved. Efficacy is markedly superior to a clinical stage CAR analog, comprising an *NKG2D-CD3ζ* fusion. Structure-function analysis using an extended CAR panel demonstrates that potency is dependent on membrane proximity of signaling units, high NKG2D cell surface expression, adaptor structure, provision of exogenous Dap10, and inclusion of one rather than three immune tyrosine activation motifs per signaling unit. Potent therapeutic impact of *NKG2D/Dap10-12* T cells is also underpinned by enhanced oxidative phosphorylation, reduced senescence, and transcriptomic re-programming for increased ribosomal biogenesis.

INTRODUCTION

NKG2D is expressed by natural killer (NK) cells, CD8$^+$ αβ T cells, γδ T cells, invariant NKT cells, and some CD4$^+$ T cell subsets.[1] It mediates the recognition of cells that express any of the eight NKG2D ligands (NKG2DLs), namely MHC class I polypeptide-related sequence (MIC)A/B and UL16-binding protein (ULBP) 1–6. NKG2DLs are widely expressed in transformed cells owing to several pathological processes, including DNA damage, replicative and oxidative stress, hypoxia, oncogene expression, the unfolded protein response, senescence, and epithelial-to-mesenchymal transition.[1] They are also found in immunosuppressive leukocytes and endothelial cells within the tumor microenvironment.[1] Although chronic inflammation involving NKG2D can sometimes accelerate tumorigenesis,[2] this receptor plays a key role in the elimination of malignant cells.[1] Consequently, NKG2D-deficient mice are more susceptible to spontaneous tumor formation in several models.[3] However, this immune surveillance pathway is compromised in advanced cancer owing to NKG2D downregulation by shed NKG2DL and immunosuppressive factors such as transforming growth factor β.[1]

Immunotherapy using modular synthetic fusion receptors known as chimeric antigen receptors (CARs) has achieved profound impact against selected hematological malignancies.[4] To target NKG2DL in diverse cancers, several NKG2D-based CARs have been developed.[5–12] The prototype developed by Sentman et al. consists of a fusion of the CD3ζ endodomain to NKG2D and has undergone extensive clinical development by Celyad Oncology as CYAD-01. Although autologous CYAD-01 has proven to be well tolerated, efficacy has been limited.[13–15] A major challenge is the fact that NKG2DLs are expressed on activated T cells, compromising CAR T cell expansion and function.[16] A similar phenomenon has been described using a

© 2024 The Author(s). Published by Elsevier Inc.
This is an open access article under the CC BY license (http://creativecommons.org/licenses/by/4.0/).

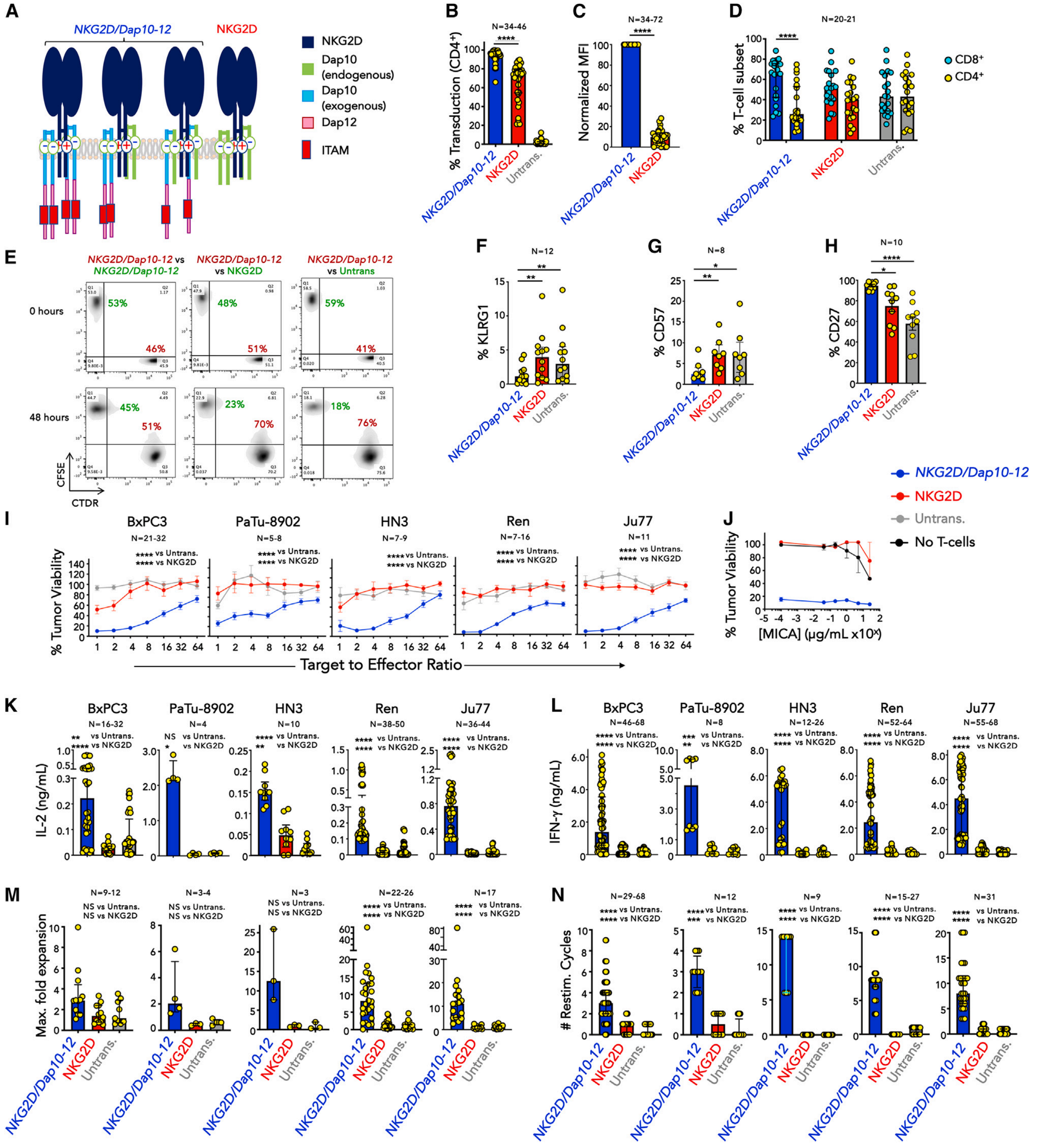

Figure 1. *In vitro* characterization of *NKG2D/Dap10-12* CAR T cells

(A) Structure of *NKG2D/Dap10-12* CAR, making comparison with the NKG2D/Dap10 complex. NKG2D encoded by the *NKG2D/Dap10-12* vector is also expected to associate with endogenous Dap10 present in T cells, giving rise to additional complexes, two of which are shown.

(B) Flow cytometric analysis of NKG2D expression in $CD4^+$ T cells following transduction with the *NKG2D/Dap10-12* CAR or NKG2D alone, making comparison with untransduced cells (median + interquartile range). Number of biological replicates is indicated on this and subsequent panels. ****$p < 0.0001$ (Kruskal-Wallis).

(C) Normalized MFI of NKG2D expression in $CD4^+$ T cells following transduction with the *NKG2D/Dap10-12* CAR or NKG2D alone. Data (median + interquartile range) were normalized to expression in *NKG2D/Dap10-12* CAR T cells (set to 100). ****$p < 0.0001$ (Mann-Whitney).

(D) CD4/CD8 analysis on day 10 of culture (median + interquartile range). ****$p < 0.0001$ (Mann-Whitney).

(legend continued on next page)

Lym-1-specific CAR containing a 4-1BB + CD3ζ endodomain.[17] In that setting, substitution of a Dap10-Dap12 endodomain helped to overcome this issue.

The linear nature of CYAD-01 and all clinically approved CARs contrasts with several naturally occurring multichain immune recognition receptors, which signal via non-covalently linked adaptors. Owing to their intrinsically unstructured nature, adaptor signaling units are believed to engage partners with enhanced speed and versatility.[18] While adaptor-based CARs have been understudied, proof of principle has been demonstrated by the co-expression of Dap12 with a single-chain antibody-killer cell immunoglobulin-like receptor (KIR)2DS2 fusion receptor.[19]

Here, we have evaluated a variety of linear and adaptor NKG2D-based CAR designs that contain Dap10+Dap12, making comparison with traditional CD3ζ- and CD28-based architectures. We report that profound and durable efficacy together with functional CAR T cell persistence is achieved in a range of challenging solid tumor models using NKG2D-containing adaptor CARs in which signaling is provided by Dap10 and a single immunoreceptor tyrosine-based activation motif (ITAM) source, notably Dap12.

RESULTS

Expression of the *NKG2D/Dap10-12* adaptor CAR in human T cells

To target NKG2DL, an adaptor CAR termed *NKG2D/Dap10-12* was engineered by co-expressing NKG2D with a fusion of full-length Dap10 joined to the Dap12 endodomain (Figure 1A). To standardize nomenclature, CAR names list all constituent components (e.g., *NKG2D/Dap 10-12*) and are italicized. A forward slash denotes an adaptor association between the targeting moiety (NKG2D) and the signaling unit (Dap10-12), while a dash signifies a direct fusion of two components (Dap10-12). We envisioned that this adaptor design would simulate a second-generation CAR that delivers an activating signal (via Dap12) and a co-stimulatory signal (via Dap10). Since NKG2D can associate with endogenous Dap10, some additional complexes that are predicted to form in *NKG2D/Dap10-12*-engineered T cells are also illustrated (Figure 1A).

To examine cell surface expression of *NKG2D/Dap10-12*, comparison was made with control human T cells that were untransduced (untrans.) or engineered to over-express NKG2D alone. Following retroviral delivery, high-level cell surface NKG2D expression was detected in both *NKG2D/Dap10-12*-transduced $CD4^+$ and $CD8^+$ T cells (Figure S1A). Unlike $CD4^+$ cells, $CD8^+$ T cells also express endogenous NKG2D (Figure S1A). Consequently, transduction efficiency was quantified as percentage cell surface NKG2D expression in $CD4^+$ T cells.

To confirm that the *NKG2D/Dap10-12* CAR consists of the predicted adaptor complex, HEK 293T cells were transfected with plasmids encoding for NKG2D alone or co-expressed with Dap10-12. While NKG2D alone accumulated intracellularly (Figure S1B), it was stabilized on the cell surface when co-expressed with Dap10-12 (Figure S1B), consistent with previous studies in NK cells[20] and T cells.[21] Homogeneous membranous co-expression of NKG2D, Dap10, and Dap12 was observed in *NKG2D/Dap10-12* CAR T cells using total internal reflection fluorescence (TIRF) microscopy (Figure S1C). Using flow cytometry, cell surface expression of additional Dap10 was confirmed (Figure S1D, upper) while Dap12 was detected intracellularly in *NKG2D/Dap10-12* CAR T cells (Figure S1D, lower). Western blotting of *NKG2D/Dap10-12* transiently transfected 293T cells demonstrated bands of the expected size for NKG2D or Dap10-12 (Figure S1E).

The proportion of transduced $CD4^+$ T cells was consistently higher in *NKG2D/Dap10-12* than $NKG2D^+$ cultures (Figure 1B). Cell surface CAR expression was expressed as normalized NKG2D mean fluorescence intensity (MFI), whereby *NKG2D/Dap10-12* was set at 100 arbitrary units (Figure 1C). Intensity of cell surface NKG2D expression in *NKG2D/Dap10-12* CAR T cells was markedly higher than that in $NKG2D^+$ cultures.

NKG2D/Dap10-12 CAR T cells enrich in culture due to fratricide

Following expansion, *NKG2D/Dap10-12* cells showed a trend toward elevated LAG-3 and Tim-3 expression, although CD69

(E) *NKG2D/Dap10-12* CAR T cells were labeled with CDTR and incubated with an equal number of CFSE-labeled T cells that expressed *NKG2D/Dap10-12*, NKG2D or were untransduced. Proportions of these cells remaining in the co-culture were analyzed by flow cytometry after 48 h (representative of three independent replicates).

(F) Expression of KLRG1 (median + interquartile range) on *NKG2D/Dap10-12* CAR T cells, making comparison with control cells that were untransduced or in which NKG2D alone was over-expressed. **$p < 0.01$ (Friedman test).

(G) Expression of CD57 (median + interquartile range) on the same T cell populations. *$p < 0.05$; **$p < 0.01$ (Friedman test).

(H) Expression of CD27 (mean ± SEM) on the same T cell populations. *$p < 0.05$; ****$p < 0.0001$ (one-way ANOVA).

(I) Cytotoxicity assays were carried out in which the indicated T cells were co-cultivated for 24 h at the specified target to effector ratio with listed tumor cell lines. Tumor cell viability was determined using a 3-(4,5-dimethylthiazol-2-yl)-2,5-diphenyl-2H-tetrazolium bromide (MTT) assay (mean ± SEM). ****$p < 0.0001$ (two-way ANOVA).

(J) Indicated T cells were co-cultivated for 24 h at a 1:1 target to effector ratio with BxPC3 tumor cells, supplemented with increasing concentrations of recombinant MICA (mean ± SEM, $n = 2$). Tumor cell viability was determined as in (I). Data are representative of three independent replicates.

(K) IL-2 concentration was analyzed in supernatants collected after 24 h from co-cultures of indicated tumor cells and T cells (target to effector ratio 1:1; median + interquartile range). *$p < 0.05$; **$p < 0.01$; ****$p < 0.0001$; NS - not significant (Kruskal-Wallis).

(L) IFN-γ concentration was analyzed in supernatants collected after 72 h from co-cultures described in (K) (median + interquartile range). **$p < 0.01$; ***$p < 0.001$; ****$p < 0.0001$ (Kruskal-Wallis).

(M) Maximum (Max.) fold expansion of indicated T cells following twice weekly re-stimulation on specified tumor cell lines (median + interquartile range). ****$p < 0.0001$; NS, not significant (Kruskal-Wallis).

(N) Number of effective re-stimulation (restim.) cycles achieved by indicated T cells when re-stimulated twice weekly on specified tumor cell lines. Re-stimulations were considered successful if <60% of tumor cells remained viable (median + interquartile range). ***$p < 0.001$; ****$p < 0.0001$ (Kruskal-Wallis). See Figures S1–S4 for additional data.

and PD1 levels remained similar to controls (Figures S2A and S2B). Moreover, cultures were significantly skewed toward the CD8$^+$ T cell subset, unlike controls (Figure 1D). This led us to hypothesize that *NKG2D/Dap10-12*$^+$ T cells undergo fratricide due to stimulation by NKG2DL, expressed by a subset of activated T cells (Figure S3A). To test this, equal numbers of CellTracker Deep Red-labeled *NKG2D/Dap10-12* cells were co-cultured with carboxyfluorescein succinimidyl ester (CFSE)-labeled *NKG2D/Dap10-12*, NKG2D, or untransduced T cells. Compared to baseline, the relative percentage of *NKG2D/Dap10-12*$^+$ cells only increased when cultured with NKG2D$^+$ or untransduced T cells (Figure 1E). *NKG2D/Dap10-12*$^+$ cells also expressed reduced NKG2DL when compared to untransduced cells (Figure S3A). Both of these findings are consistent with fratricide-mediated enrichment. *NKG2D/Dap10-12* cells retained polyfunctionality (Figure S2C), expressed significantly reduced KLRG1 (Figure 1F) and CD57 (Figure 1G), maintained elevated CD27 (Figure 1H), and exhibited predominantly effector memory differentiation (Figure S2D).

NKG2D/Dap10-12 adaptor CAR T cells mediate potent anti-tumor immunity *in vitro*

To test efficacy, we selected a panel of NKG2DL$^+$ cell lines representative of pancreatic cancer (BxPC3, PaTu-8902), head and neck cancer (HN3), and malignant pleural mesothelioma (Ren, Ju77) (Figure S3B). Consistent dose-dependent tumor cell killing was mediated by *NKG2D/Dap10-12* T cells, in contrast to untransduced or NKG2D$^+$ controls (Figure 1I). Cytolytic activity was not inhibited by soluble MICA, even at supraphysiological concentrations (Figure 1J).[22–24] Activated *NKG2D/Dap10-12* T cells produced interleukin (IL)-2 (Figure 1K) and interferon (IFN)-γ (Figure 1L) and expanded (Figure 1M) and maintained cytotoxic activity (Figure 1N) when re-stimulated on tumor cell monolayers. Moreover, maximum fold expansion of these re-stimulated CAR T cells correlated strongly with the number of re-stimulation cycles (Figure S2E, representative re-stimulation assays shown in Figures S2F–S2J).

A limitation of the aforementioned comparison is the large difference in MFI of cell surface NKG2D between *NKG2D/Dap10-12*$^+$ and NKG2D$^+$ T cells (Figure 1C). We hypothesized that endogenous Dap10 concentration is limiting for cell surface NKG2D expression in NKG2D$^+$ T cells. When NKG2D was co-expressed with Dap10 (NKG2D/Dap10; Figure S4A), cell surface NKG2D MFI was markedly upregulated, approximating closely to *NKG2D/Dap10-12* CAR T cells (Figure S4B). However, NKG2D/Dap10$^+$ T cells did not achieve enhanced tumor re-stimulation (Figures S4C and S4D) or cytolytic activity (Figures S4E and S4F), indicating that MFI was not a satisfactory explanation for differences in anti-tumor function.

NKG2D/Dap10-12 adaptor CAR T cells exert durable control of solid tumor xenografts

Next, we evaluated *in vivo* anti-tumor activity of *NKG2D/Dap10-12*$^+$ T cells using three intraperitoneal (i.p.) tumor models (Figure S3B). Intraperitoneal tumor formation provides a convenient model of some malignant tumor types, including epithelial ovarian cancer, malignant peritoneal mesothelioma, and metastatic spread of pancreatic carcinoma to the peritoneal cavity. Following i.p. CAR T cell delivery, disease control was achieved in ovarian (SKOV-3; Figure 2A), pancreatic (BxPC3 - Figure 2B), and mesothelioma cancer models (H226, Figure 2C). Ten of 18 mice (56%) achieved a sustained complete response (CR). In the H226 model, a decline in tumor burden was also observed in the NKG2D control group. This may have been due to alloreactivity of the T cells and/or co-stimulatory activity (Figure 2C). Nonetheless, tumors remained detectable in these mice.

To test functional persistence of *NKG2D/Dap10-12* CAR T cells, tumor cells were re-injected i.p. in the nine BxPC3 and H226-engrafted mice that had achieved sustained CAR T cell-induced CR. In 3/4 (BxPC3) and 5/5 cases (H226), secondary tumor inoculation was completely rejected (Figures 2B and 2C). Despite the inclusion of tumor re-challenge, *NKG2D/Dap10-12*-treated mice had a significant survival advantage in all three models, durable beyond 150 days in many cases (Figures 2D–2F). This demonstrates that *NKG2D/Dap10-12*$^+$ T cells achieve profound *in vivo* anti-tumor activity and sustained functional persistence.

NKG2D/Dap10-12 CAR T cells outperform a clinical stage NKG2D CAR

Given its extensive clinical evaluation, we next compared an analog of the NKG2D-based CYAD-01 CAR (here referred to as *NKG2D-CD3ζ*; Figure 3A) with the *NKG2D/Dap10-12* adaptor CAR. Since *NKG2D-CD3ζ* associates with endogenous Dap10, it also has potential to deliver co-stimulation and thereby act as a second-generation CAR.[25] High-efficiency expression of the *NKG2D-CD3ζ* CAR by T cells was consistently achieved (Figure 3B). This is likely due to fratricide-mediated enrichment of transduced cells, as described[16] (Figure 1E). However, MFI of *NKG2D-CD3ζ* expression was significantly lower than that of *NKG2D/Dap10-12* (Figure 3C; Figure S1A). We hypothesized that endogenous Dap10 was also a limiting factor for cell surface expression of *NKG2D-CD3ζ*. Co-expression of exogenous Dap10 with this CAR (*NKG2D-CD3ζ/Dap10*; Figure 3A) significantly boosted cell surface CAR MFI, albeit not to the level of *NKG2D/Dap10-12* (Figure 3C).

Unpredictable expansion of CYAD-01 CAR T cells owing to fratricide has been described.[16] We also found that *NKG2D-CD3ζ* CAR T cell viability (Figure S5A) and yield (Figure S5B) were both significantly reduced compared to untransduced T cells. Neither issue was seen with *NKG2D/Dap10-12* cells (Figures S5A and S5B). We observed that levels of NKG2DL (Figure S5C), CD69 (CD4$^+$ and CD8$^+$ T cells; Figures S5D and S5E), and CD25 (CD4$^+$ T cells only; Figure S5F) were all significantly higher on *NKG2D-CD3ζ* compared to *NKG2D/Dap10-12* CAR T cells. These data indicate that removal of senescent (e.g., NKG2DL)-expressing T cells is less efficient in *NKG2D-CD3ζ* cultures, resulting in a more activated phenotype. Given that Dap10 over-expression failed to correct the issue of unpredictable yield (*NKG2D-CD3ζ/Dap10* CAR; Figure S5B), these findings suggest that this is due to incorporation of CD3ζ rather than Dap12 in the CAR endodomain.

When *in vitro* anti-tumor activity of *NKG2D/Dap10-12*, *NKG2D-CD3ζ*, and *NKG2D-CD3ζ/Dap10* CAR T cells was compared using BxPC3 and Ren cell lines, the number of

CellPress
OPEN ACCESS

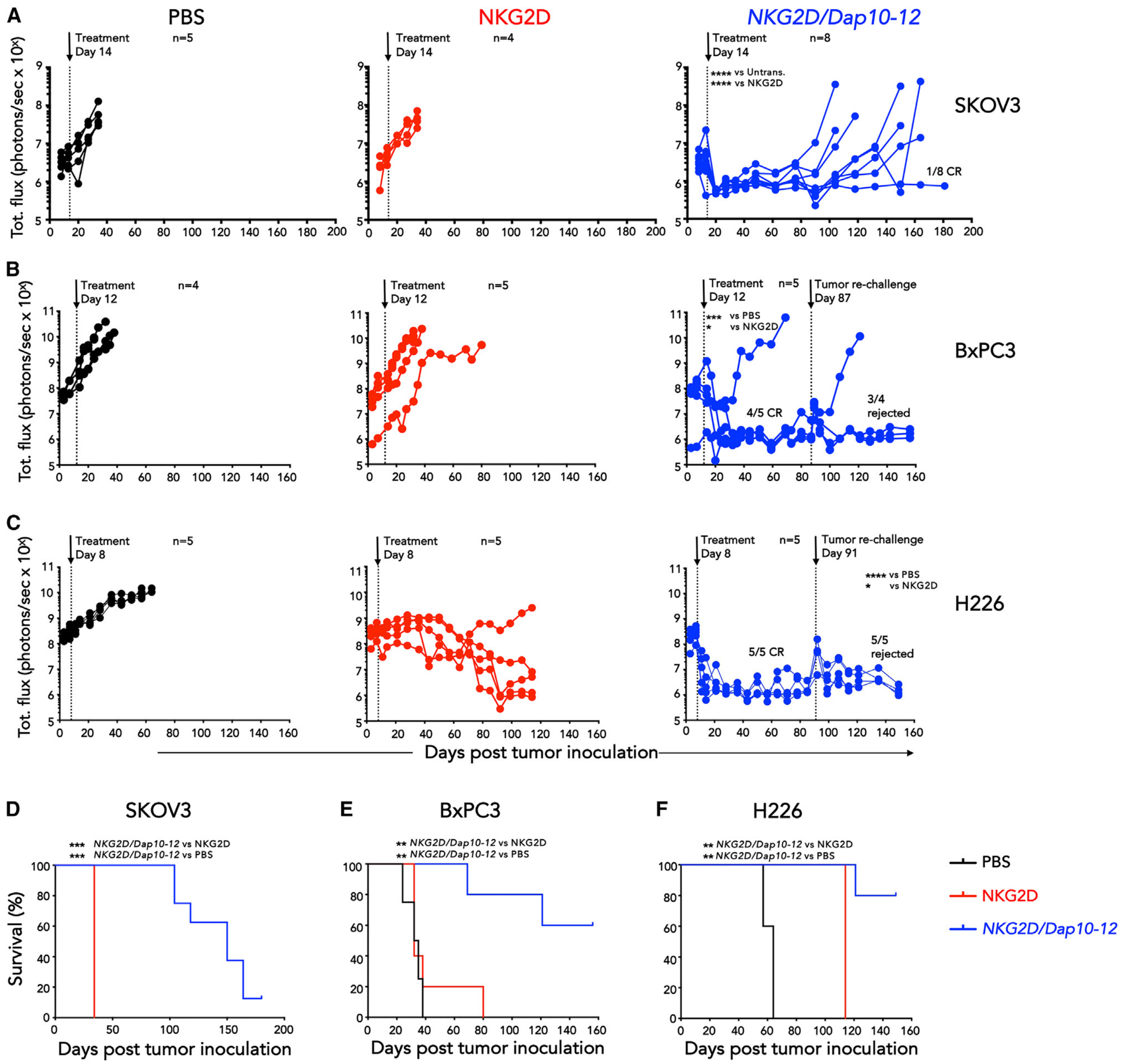

Figure 2. *In vivo* anti-tumor activity of *NKG2D/Dap10-12* CAR T cells

(A) Firefly luciferase (ffLuc)-expressing SKOV-3 cells (5×10^5 cells) were established i.p. in female NSG mice for 14 days prior to i.p. administration of 10 million of the indicated T cell populations, or PBS. Tumor burden was monitored using bioluminescence imaging (BLI), and complete response (CR) numbers are indicated. Number of biological replicates is indicated on this and subsequent panels. ****$p < 0.0001$ (two-way ANOVA post-treatment to day 34).

(B) ffLuc-expressing BxPC3 cells (1×10^5 cells) were established i.p. in NSG mice for 12 days prior to i.p. administration of 10 million of the indicated T cell populations, or PBS. Tumor burden was monitored using BLI, and CR numbers are indicated. *$p < 0.05$; ***$p < 0.001$ (two-way ANOVA post-treatment to day 32). Re-challenge with a similar dose of BxPC3 cells was performed in tumor-free mice on day 87. Rejection frequency is indicated.

(C) ffLuc-expressing H226 mesothelioma cells (1×10^6 cells) were established i.p. in NSG mice for 8 days prior to i.p. administration of 10 million of the indicated T cell populations, or PBS. Tumor burden was monitored using BLI, and CR numbers are indicated. *$p < 0.05$; ****$p < 0.0001$ (two-way ANOVA post-treatment to day 52). Re-challenge with a similar dose of H226 cells was performed in tumor-free mice on day 91. Rejection frequency is indicated.

(D–F) Survival of mice described in (A–C), respectively. **$p < 0.01$; ***$p < 0.001$ (Log rank/Mantel-Cox).

productive tumor re-stimulation cycles (Figure 3D) and cytokine production (Figures 3E and 3F) tended to be greater in *NKG2D/Dap10-12* cultures. This was not attributable to differences in activation-induced cell death (Figure S5G).

To compare *in vivo* anti-tumor activity of these CARs, the previously described i.p. BxPC3 pancreatic xenograft model (Figure 2B) was used. A lower CAR T cell dose of 4 million cells was used on this occasion. While efficacy of both

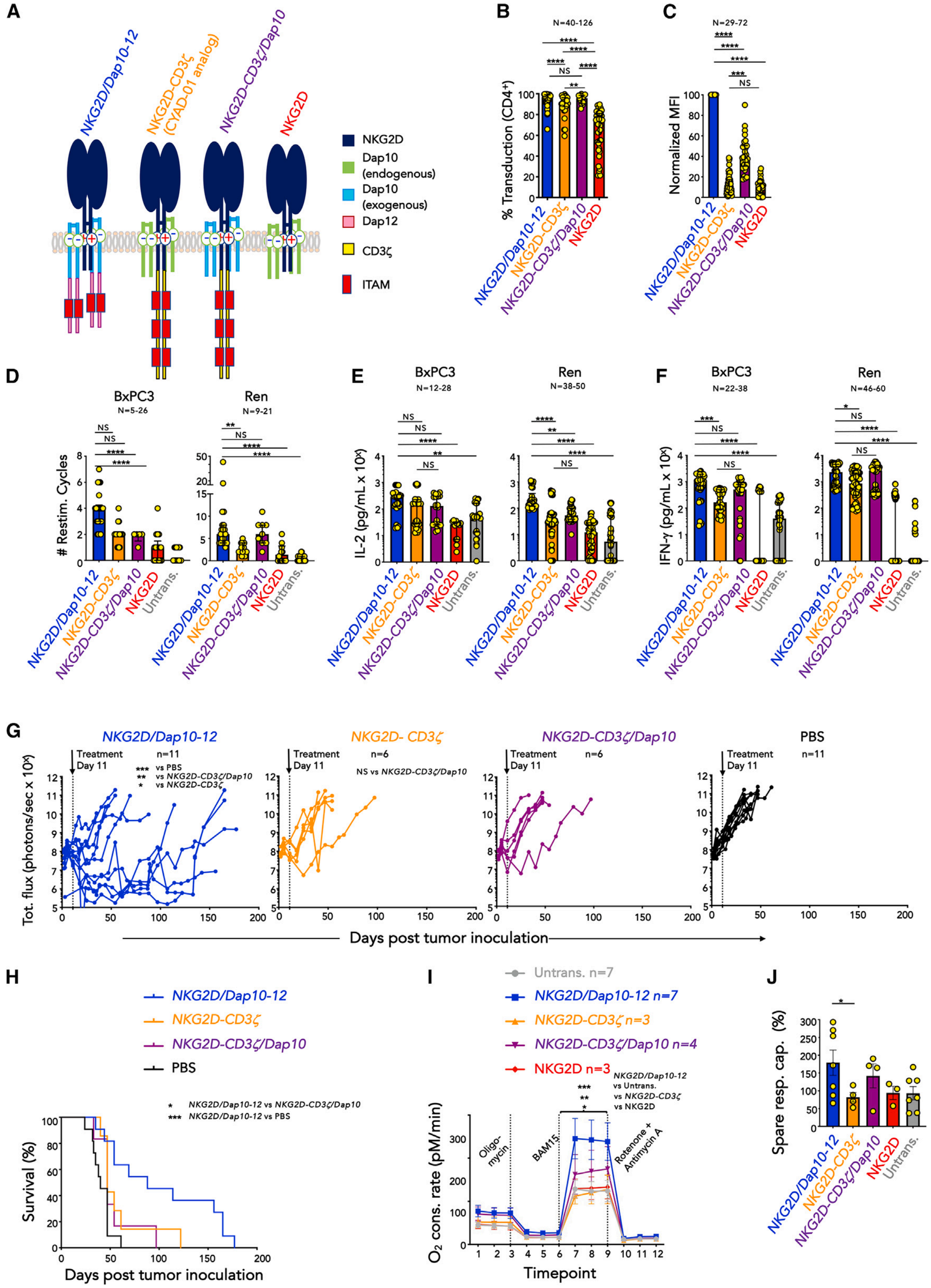

(legend on next page)

NKG2D-CD3ζ and *NKG2D-CD3ζ/Dap10* was poor, sustained tumor control was observed in 6/11 *NKG2D/Dap10-12*$^+$ T cell-treated mice (Figure 3G) accompanied by increased survival (Figure 3H).

A key determinant of CAR T cell efficacy is mitochondrial fitness.[26,27] Seahorse analysis showed that *NKG2D/Dap10-12* T cell cultures had significantly greater maximal respiratory capacity compared to *NKG2D-CD3ζ* cells, while *NKG2D-CD3ζ/Dap10* CAR T cells had an intermediate capacity (Figure 3I). Additionally, there was an increase in spare respiratory capacity in *NKG2D/Dap10-12* T cell cultures (Figure 3J), consistent with enhanced oxidative phosphorylation capacity.

We next extended our *in vivo* comparison of *NKG2D/Dap10-12* to the clinical stage CAR analog, *NKG2D-CD3ζ*. In a subcutaneous (s.c.) mesothelioma patient-derived xenograft model (PDX #008), intravenous (i.v.) administration of 10 million *NKG2D-CD3ζ*- or NKG2D-engineered T cells on day 108 was completely ineffective (Figure 4A). Strikingly, by contrast, *NKG2D/Dap10-12* T cells led to disease eradication in 6/7 mice (Figure 4A). This was especially noteworthy given the relatively modest expression of NKG2DL on this tumor (Figure S3B). When treatment dose was reduced to 4 million cells and administration further delayed to day 127, a significant delay in tumor progression was still evident in *NKG2D/Dap10-12*-treated mice (Figure S6).

Comparison was next undertaken in two ovarian cancer xenograft models: Kuramochi and A2780. Combined i.p. and i.v. CAR T cell dosing was utilized, as undertaken in a recent clinical trial.[28] Complete Kuramochi tumor eradication was achieved in 7/9 mice using *NKG2D/Dap10-12*, but not *NKG2D-CD3ζ* T cells (Figure 4B). By contrast, both CARs proved highly effective in the A2780 model, eliciting 3/4 (*NKG2D-CD3ζ*) and 7/7 (*NKG2D/Dap10-12*) CRs (Figure 4C). However, when re-challenge was performed in either Kuramochi or A2780 mice, tumor rejection occurred in the *NKG2D/Dap10-12* (5/7 Kuramochi and 7/7 A2780 mice) but not the *NKG2D-CD3ζ* group (0 of 3 A2780 mice; Figures 4B and 4C). While human CAR T cell persistence in non-obese diabetic severe combined immunodeficient common gamma chain null (NSG) mice may be boosted by xenoreactivity against mouse major histocompatibility complex antigens, this cannot fully explain these findings since efficacy of *NKG2D/Dap10-12* was not matched by *NKG2D-CD3ζ*. Despite the inclusion of tumor re-challenge, survival of *NKG2D/Dap10-12*-treated mice was also significantly prolonged in all models (Figures 4D–4F). These data emphasize that *NKG2D/Dap10-12* CAR T cells mediate sustained efficacy and persistence in several solid tumor models.

Evaluation of the role of CAR structure in targeting of NKG2DLs

We next undertook a structure-function analysis of NKG2D-based CAR T cell function using an extended CAR panel (nomenclature/numbering and predicted structure; Figure 5A). Normalized MFI of cell surface CAR expression is depicted in Figure 5B, in which *NKG2D/Dap10-12* has been set to 100 units for each donor. Tumor re-stimulation assays were undertaken on Ren monolayers to model antigen-induced exhaustion.[26,29] Comparison was made between CAR pairs and pooled CAR groups that were identical except for a single structural attribute. First, we compared full-length versus endodomain-truncated NKG2D (N(Tr.)). Inclusion of an NKG2D endodomain places fused signaling modules farther from the plasma membrane, which compromises CD28 and 4-1BB co-stimulatory function.[26,30] In pairwise analysis, we observed greater re-stimulation activity of N(Tr.) CARs (Figure 5C), a finding that became significant when data from all full-length and matched N(Tr.) CARs were pooled (Figure 5D). Second, we studied the role of ITAM number per CAR monomer. We compared CD3ζ (3 ITAMs per unit) with two single ITAM options, namely Dap12 and CD3ζ(1XX), in which ITAMs 2 and 3 were inactivated by mutation.[31,32] A trend toward enhanced function of single ITAM CARs was noted in pairwise analysis, with greater significance when pooled CAR groups were compared (Figures 5E and 5F). Third, we evaluated the incorporation of identical signaling units in either a linear or an adaptor CAR format and found that the latter achieved greater tumor re-stimulation activity (Figures 5G and 5H). Fourth, we tested the impact of expression of additional Dap10 within the CAR vector and observed that this also resulted in greater re-stimulation capacity (Figures 5I and 5J). Fifth, we studied CARs with a normalized NKG2D MFI above or below the median value

Figure 3. Anti-tumor activity of adaptor and linear NKG2D-based CAR T cells

(A) Structure of *NKG2D-CD3ζ* (analog of the clinical stage CYAD-01 CAR), *NKG2D-CD3ζ/Dap10*, *NKG2D/Dap10-12* CARs, and NKG2D.

(B) Flow cytometric analysis of NKG2D expression in CD4$^+$ T cells 10 days following transduction with the indicated CARs or NKG2D alone (median + interquartile range). Number of biological replicates is indicated on this and subsequent panels. $^{**}p < 0.01$; $^{****}p < 0.0001$; NS, not significant (Kruskal-Wallis).

(C) Normalized MFI of NKG2D expression in CD4$^+$ T cells 10 days following transduction with the indicated CARs or NKG2D alone (median + interquartile range). Data were normalized in each experiment to expression in *NKG2D/Dap10-12* CAR T cells, which was set to 100. $^{***}p < 0.001$; $^{****}p < 0.0001$; NS, not significant (Kruskal-Wallis).

(D) Number of twice weekly re-stimulation (restim.) cycles achieved by indicated T cells on specified tumor cell lines (median + interquartile range). $^{**}p < 0.01$; $^{****}p < 0.0001$; NS, not significant (Kruskal-Wallis test).

(E) IL-2 concentration was analyzed in supernatants collected after 24 h from co-cultures of indicated tumor cells and T cells (target to effector ratio 1:1; median + interquartile range). $^{**}p < 0.01$; $^{****}p < 0.0001$; NS, not significant (Kruskal-Wallis).

(F) IFN-γ concentration was analyzed in supernatants collected after 72 h from co-cultures described in E (median + interquartile range). $^{*}p < 0.05$; $^{***}p < 0.001$; $^{****}p < 0.0001$; NS, not significant (Kruskal-Wallis).

(G) ffLuc-expressing BxPC3 cells (1×10^5 cells) were established i.p. in NSG mice for 11 days prior to i.p. administration of 4 million of the indicated T cell populations, or PBS. Tumor burden was monitored using BLI. $^{*}p < 0.05$; $^{**}p < 0.01$; $^{***}p < 0.001$; NS, not significant (two-way ANOVA post-treatment).

(H) Survival of mice described in (G). $^{*}p < 0.05$; $^{***}p < 0.001$ (Log rank/Mantel-Cox).

(I) Seahorse mitochondrial stress testing of indicated T cells, adding specified inhibitors as indicated (mean ± SEM, $n = 3–7$). $^{*}p < 0.05$; $^{**}p < 0.01$; $^{***}p < 0.001$ (two-way ANOVA, comparing *NKG2D/Dap10-12* CAR T cells to the indicated comparator group at time points 7–9).

(J) Spare respiratory (resp.) capacity of T cells described in (I) (mean ± SEM, $n = 3–7$). $^{*}p < 0.05$ (unpaired t test). See Figure S5 for additional data.

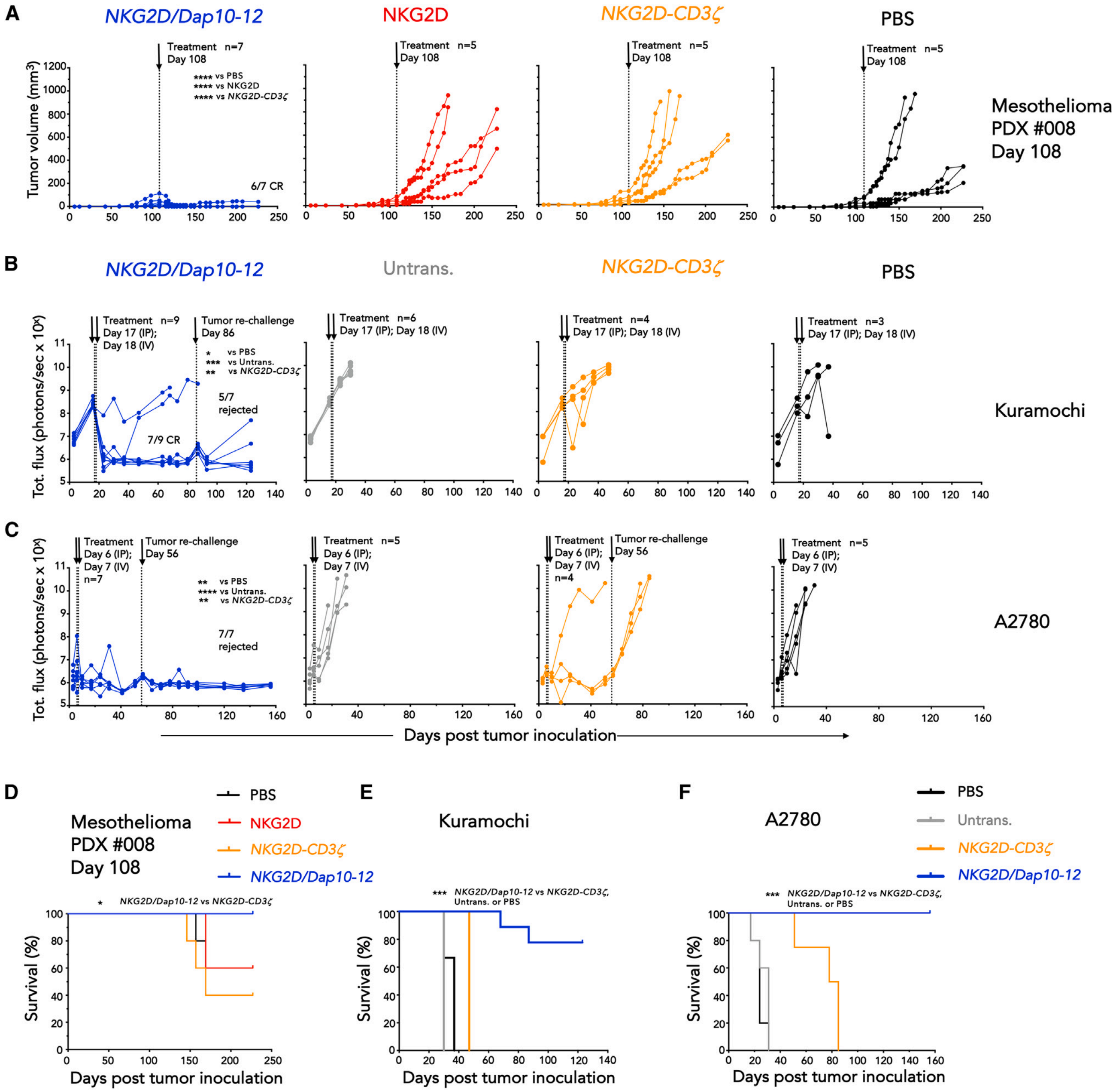

Figure 4. ***In vivo* comparison of *NKG2D/Dap10-12* and *NKG2D-CD3ζ* CARs**

(A) Mesothelioma PDX_008 was engrafted s.c. in NSG mice. After 108 days 10 million of the indicated T cell populations or PBS were injected i.v. Tumor volume was monitored by caliper measurements. Number of biological replicates and CRs are indicated on this and subsequent panels. ****$p < 0.0001$ (two-way ANOVA post-treatment).

(B) ffLuc-expressing Kuramochi (1×10^5 cells) were established i.p. in NSG mice for 17 days prior to i.p. administration of 5 million of the indicated T cell populations, or PBS. A second dose of 5 million T cells or PBS was administered i.v. on day 18. Tumor burden was monitored using BLI, and CR numbers are indicated. *$p < 0.05$; **$p < 0.01$; ***$p < 0.001$ (two-way ANOVA post-treatment). Re-challenge with a similar dose of Kuramochi cells was performed in tumor-free mice on day 86. Rejection frequency is indicated.

(C) ffLuc-expressing A2780 (1×10^5 cells) were established i.p. in NSG mice for 6 days prior to i.p. administration of 2 million of the indicated T cell populations, or PBS. A second dose of 2 million T cells or PBS was administered i.v. on day 7. Tumor burden was monitored using BLI, and CR numbers are indicated. **$p < 0.01$; ****$p < 0.0001$ (two-way ANOVA post-treatment). Re-challenge with a similar dose of A2780 cells was performed in tumor-free mice on day 56. Rejection frequency is indicated.

(D–F) Survival of mice described in (A–C), respectively. *$p < 0.05$; ***$p < 0.001$ (Log rank/Mantel-Cox). See Figure S6 for additional data.

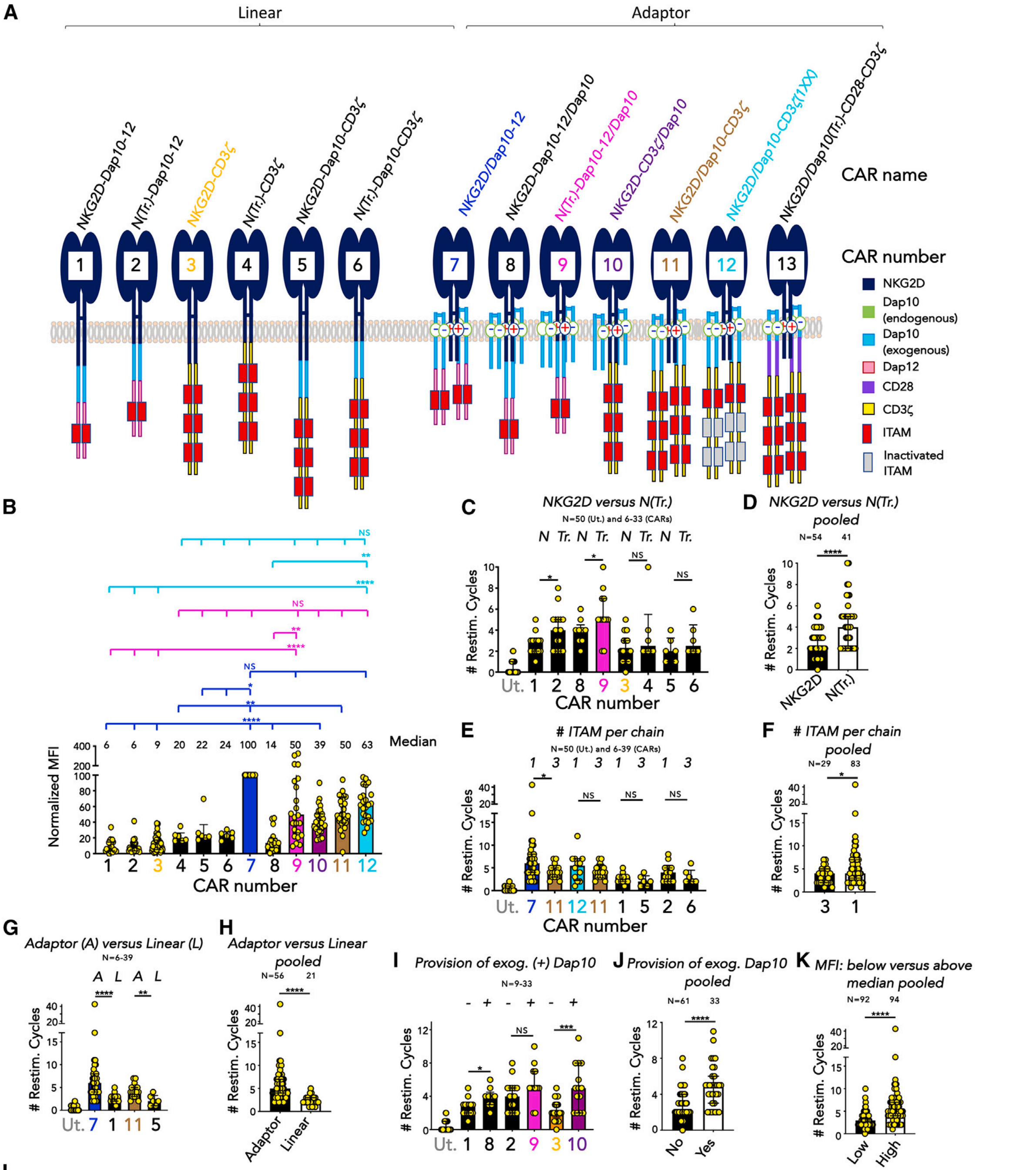

L

CAR	1	2	3	4	5	6	7	8	9	10	11	12	13
Membrane proximity of signaling units	0	1	0	1	0	1	1	0	1	0.5	1	1	1
Single ITAM per signaling unit	1	1	0	0	0	0	1	1	1	0	0	1	0
Adaptor format	0	0	0	0	0	0	1	1	1	1	1	1	1
Inclusion of exogenous Dap10	0	0	0	0	0	0	1	1	1	1	1	1	0
High MFI	0	0	0	0	0	0	1	0	1	1	1	1	1
Dap10 rather than CD28	1	1	0	0	1	1	1	1	1	1	1	1	0
**Combined score**	**2**	**3**	**0**	**1**	**1**	**2**	**6**	**4**	**6**	**4.5**	**5**	**6**	**3**
Advanced to in vivo study			X				X		X		X	X	

(legend on next page)

of 29.4. Re-stimulation cycle number was significantly greater in the high-MFI compared to low-MFI CAR group (Figure 5K). Sixth, we compared the delivery of co-stimulation by Dap10 versus CD28 (Figure S7). Using the *NKG2D/Dap10-CD3ζ* adaptor CAR as a starting point, Dap10 endodomain sequences were removed and substituted with those from CD28 (*NKG2D/Dap10(Tr.)-CD28⁻CD3ζ*, Figures 5A and S7A). Transduction efficiency (Figure S7B) and cell surface CAR MFI (Figure S7C) were both significantly higher for *NKG2D/Dap10-CD3ζ* compared to *NKG2D/Dap10(Tr.)-CD28⁻CD3ζ*. Moreover, *NKG2D/Dap10-CD3ζ* T cells underwent more productive re-stimulation cycles on Ren monolayers (Figure S7D) accompanied by sustained tumor cytolytic activity (Figure S7E).

In conclusion, these data indicate that optimal function of NKG2D-based CARs is dependent on positioning of fused signaling units close to the plasma membrane, high CAR MFI, co-stimulation by Dap10 rather than CD28, provision of exogenous Dap10 to yield an adaptor CAR format, and inclusion of a single rather than triple ITAMs per CAR monomer.

In vivo comparison of anti-tumor activity of optimized NKG2D-based CARs

The extended CAR panel (Figure 5A) was scored for attributes predictive of improved anti-tumor activity (Figure 5L), and the top four candidates were advanced to *in vivo* testing. Once again, efficacy was benchmarked against the clinical stage *NKG2D-CD3ζ* CAR. Subcutaneous BxPC3 tumors were established for 17 days prior to i.v. delivery of CAR T cells. This is a more stringent model than the i.p. BxPC3 model since CAR T cells need to traffic to the site of disease. Figure 6A demonstrates that anti-tumor activity fell into two groups. Greatest tumor control (Figure 6A) and survival (Figure 6B) were seen with all CARs that contain a single ITAM per CAR monomer. By contrast, CARs that contained triple ITAMs did not confer any significant anti-tumor activity, compared to untransduced T cells. These data emphasize the importance of reduced ITAM dosage, with comparable results obtained using a single membrane-proximal ITAM from Dap12 or CD3ζ. On the other hand, high cell surface CAR expression achieved through provision of additional Dap10 in isolation did not improve efficacy, indicated by the equally poor performance of the *NKG2D/Dap10-CD3ζ* and *NKG2D-CD3ζ* CARs.

In vitro expansion of NKG2D CAR T cells is compromised by CD3ζ

To better understand the difficulty in expanding *NKG2D-CD3ζ* CAR T cells (Figure S5B), we next compared the expansion of the extended CAR T cell panel (Figure 5A). Although comparison between other individual CARs did not yield significant differences (data not shown), we noted that expansion of *NKG2D/Dap10-12* CAR T cells (Figure S5H) or all single ITAM-containing CAR T cells (Figure S5I) was significantly greater than pooled expansion of the three CD3ζ CAR T cell populations tested. This provides further evidence that the heightened NKG2DL-driven activation signal delivered by the three ITAMs in the CD3ζ endodomain is linked to the poorer viability (Figure S5A) and expansion (Figures S5B, S5H, and S5I) of *NKG2D-CD3ζ* CAR T cells.

RNA sequencing analysis

To further understand functional differences between these NKG2D-based CARs, bulk RNA sequencing was performed on CAR T cells after stimulation on immobilized human MICA for 24 h. Comparison was made between *NKG2D/Dap10-12* and three CD3ζ-based CARs (e.g., *NKG2D-CD3ζ*, *NKG2D-CD3ζ/Dap10*, and *NKG2D/Dap10-CD3ζ*). Untransduced and NKG2D over-expressing T cells served as additional controls. Principal-component analysis (PCA) demonstrated that clustering of samples was mainly driven by the presence or absence of a

Figure 5. Evaluation of structural attributes in NKG2D-based CAR function
(A) Structure, nomenclature, and numbering of an extended linear and adaptor NKG2D-based CAR panel.
(B) Normalized MFI of NKG2D expression in CD4$^+$ T cells 10 days following transduction with the indicated CARs (numbering scheme shown in A; median + interquartile range). Data were normalized to expression in *NKG2D/Dap10-12* CAR T cells (CAR number 7), which was set to 100. Numbers of biological replicates: 23 (CAR 1), 26 (2), 55 (3), 6 (4), 6 (5), 6 (6), 72(7), 17 (8), 26 (9), 29 (10), 31 (11), 26 (12). *$p < 0.05$; **$p < 0.01$; ****$p < 0.0001$; NS, not significant (Kruskal-Wallis).
(C) Number of twice weekly re-stimulation (restim.) cycles on Ren tumor cells, making comparison between otherwise structurally matched CARs that contain full length (N) or endodomain truncated (Tr.) NKG2D (NTr.). Ut, untransduced control T cells (median + interquartile range). Numbers of biological replicates: 50 (Ut), 15 (CAR 1), 17 (2), 9 (8), 12 (9), 33 (3), 6 (4), 6 (5), 6 (6). *$p < 0.05$; NS, not significant (Kruskal-Wallis).
(D) Data in (C) have been pooled, making comparison between all CARs that contain full length (N) or endodomain truncated NKG2D (NTr.; median + interquartile range). Numbers of biological replicates are indicated. ****$p < 0.0001$ (Kruskal-Wallis).
(E) Number of restim. cycles (median + interquartile range) on Ren tumor cells, making comparison between otherwise structurally matched NKG2D-based CARs that contain 1 or 3 ITAMs per CAR endodomain chain. Numbers of biological replicates: 50 (Ut), 39 (CAR 7), 17 (11), 12 (12), 17 (11), 15 (1), 6 (5), 17 (2), 6 (6). *$p < 0.05$; NS, not significant (Kruskal-Wallis).
(F) Pooled analysis of data shown in (E) (median + interquartile range). Numbers of biological replicates are indicated. *$p < 0.05$ (Kruskal-Wallis).
(G) Number of restim. cycles (median + interquartile range) on Ren tumor cells, making comparison between otherwise structurally matched NKG2D-based CARs with an adaptor (A) or linear (L) conformation. Numbers of biological replicates: 50 (Ut), 39 (CAR 7), 15 (1), 17 (11), 6 (5). ****$p < 0.0001$; **$p < 0.01$ (Kruskal-Wallis).
(H) Pooled analysis of data shown in (G) (median + interquartile range). Numbers of biological replicates are indicated. ****$p < 0.0001$ (Kruskal-Wallis).
(I) Number of restim. cycles (median + interquartile range) on Ren tumor cells achieved by NKG2D-based CAR T cells expressed alone (−) or together with exogenous Dap10 (+). Numbers of biological replicates: 50 (Ut), 15 (CAR 1), 9 (8), 17 (2), 12 (9), 33 (3), 14 (10). *$p < 0.05$; ***$p < 0.001$; NS, not significant (Kruskal-Wallis).
(J) Pooled analysis of data shown in (I) (median + interquartile range). Numbers of biological replicates are indicated. ****$p < 0.0001$ (Kruskal-Wallis).
(K) Pooled analysis of all NKG2D-based CARs shown in (A), comparing number of restim. cycles (median + interquartile range) achieved by CAR T cells with normalized MFI below or above the median value. Numbers of biological replicates are indicated. ****$p < 0.0001$ (Kruskal-Wallis).
(L) CARs shown in (A) were scored for the indicated attributes in order to select candidates for *in vivo* comparison. Although scoring poorly, CAR 3 (*NKG2D-CD3ζ*) was also advanced in light of the extensive clinical data pertaining to this CAR. See Figure S7 for additional data.

CellPress
OPEN ACCESS

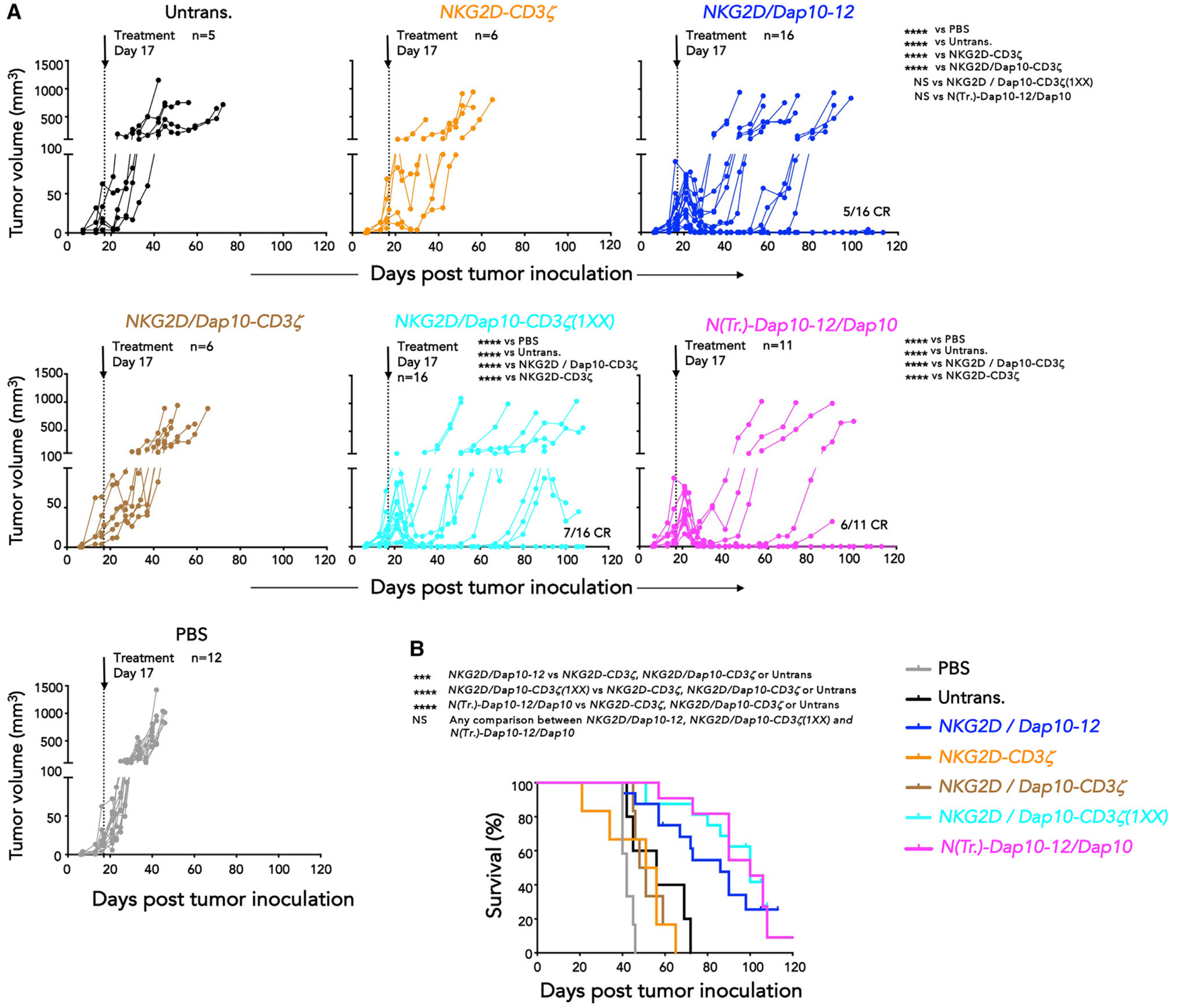

Figure 6. ***In vivo*** **comparison of optimized NKG2D-based CAR T cells in a stringent s.c. pancreatic tumor xenograft model**

(A) BxPC3 cells (1×10^5 cells) were established s.c. in NSG mice for 17 days prior to i.v. administration of 10 million of the indicated T cell populations, or PBS. Tumor volume was monitored using calipers. CR numbers are indicated. $^{****}p < 0.0001$; NS, not significant (two-way ANOVA, tumor burden post-treatment). Numbers of biological replicates are indicated.

(B) Survival of mice described in (A). $^{***}p < 0.001$; $^{****}p < 0.0001$; NS, not significant (Log rank/Mantel-Cox).

functional CAR (Figure 7A). When PCA analysis was restricted to the four functional CARs alone, sample clustering was instead driven by donor origin (Figure 7B). There were 217 differentially expressed genes (DEGs, false discovery rate [FDR] < 0.05) when *NKG2D/Dap10-12* samples were compared to control CARs (Figure 7C). Potential roles of these genes are considered further in the discussion.

Gene set enrichment analysis (GSEA) was next undertaken. Analysis of Kyoto Encyclopedia of Genes and Genomes (KEGG) gene sets revealed that *NKG2D/Dap10-12* T cells exhibited highly enriched activity of ribosome (Figure 7D) and oxidative phosphorylation signatures (Figure 7E) when compared to CD3ζ-containing CARs, in agreement with the Seahorse data. Heatmaps indicated that the majority of transcripts in both of these gene lists were increased in *NKG2D/Dap10-12* T cells (Figures 7F and 7G, respectively). Similar findings were obtained using the REACTOME and HALLMARK gene sets (Figure 7H). In addition, p53 signaling and cellular senescence pathways were both downregulated in *NKG2D/Dap10-12* T cells when compared to the other NKG2D CAR groups (Figure 7H), in agreement with flow cytometry data (Figures 1F–1H).

DISCUSSION

Our primary finding is that compact NKG2D-based adaptor CARs that employ Dap10 and a single ITAM activation source

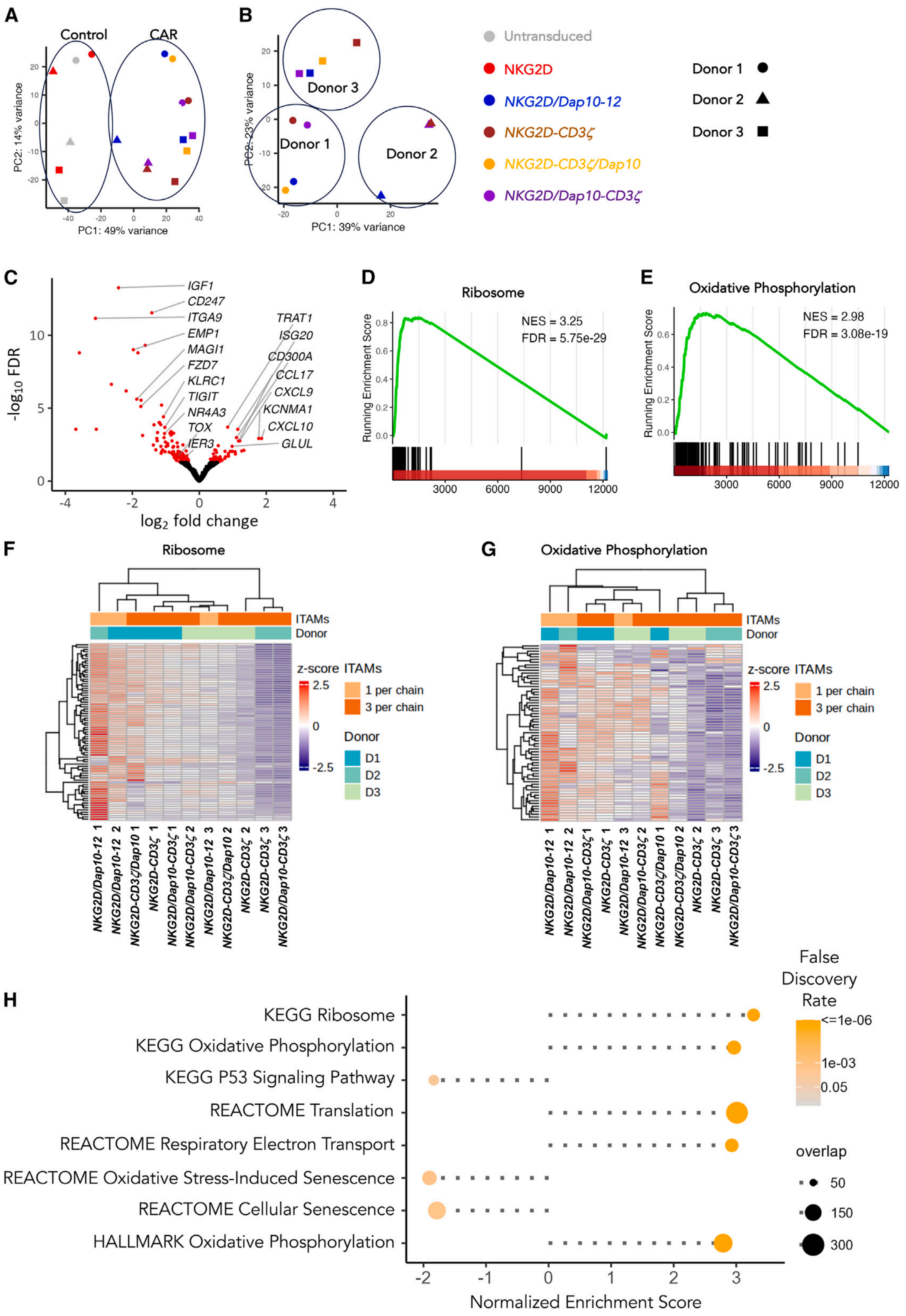

Figure 7. RNA sequencing analysis of *NKG2D/Dap10-12* CAR T cells making comparison with CD3ζ-based CAR T cells

(A) The indicated CAR and control T cell populations were stimulated for 24 h on immobilized MICA prior to RNA extraction and bulk sequencing analysis (n = 3 donors). Principal-component analysis (PCA) is shown.

(legend continued on next page)

(either Dap12 or CD3ζ(1XX)) achieve compelling efficacy across a broad range of solid tumor models. Using the best characterized of these CARs, *NKG2D/Dap10-12*, durable CRs were achieved in seven discrete xenografts representative of pancreatic cancer, ovarian cancer, and malignant pleural mesothelioma, while prolonged disease control was observed in two additional models. Importantly, CRs were maintained in many cases over the natural lifespan of the mice. Anti-tumor activity was benchmarked against an analog of the clinical stage CYAD-01 CAR (*NKG2D-CD3ζ*), which has achieved clinical responses in patients with both solid and hematological malignancies.[33] Immunotherapy using *NKG2D/Dap10-12* CAR T cells achieved markedly better efficacy in all models tested. Moreover, tumor re-challenge was rejected in 20/23 cases where *NKG2D/Dap10-12* CAR T cells had achieved CRs, demonstrating their functional persistence. Once again, this was not achieved with the CYAD-01 CAR analog in the single model in which CRs were seen.

Several factors are likely to be important in the attainment of optimal anti-tumor activity of NKG2D-based CARs. First, we found that single ITAM-containing activation modules favored enhanced efficacy. Similarly, a CD3ζ(1XX) activation domain[31,32] has previously been shown to potentiate the function of CD19-targeted CAR T cells. Second, provision of co-stimulation by Dap10 promoted greater anti-tumor activity than did CD28. Previous work has indicated that the YMNM motif in CD28 may be linked to enhanced CAR T cell exhaustion. This can be reduced by mutation of N-> F, leading to increased Akt activation and reduced activation of Vav1 and phospholipase C-γ.[34] Thus, the combined delivery of a more "calibrated" activation signal via a single ITAM and co-stimulatory signal via the distinct YXXM motif in Dap10 may be key to sustained functional persistence and anti-tumor activity. Comprehensive mutagenesis and signaling studies are required to disentangle precisely how signals delivered by the *NKG2D/Dap10-12* CAR elicit anti-tumor activity.

NKG2D/Dap10-12 CAR T cells underwent spontaneous enrichment in culture owing to fratricide, resulting in ligand-dependent signaling. CAR stimulation was triggered by exposure to low levels of NKG2DL on activated T cells. Continuous CAR signaling generally impairs CAR T cell expansion and promotes exhaustion.[35] However, expansion and anti-tumor activity of *NKG2D/Dap10-12* CAR T cells were preserved nonetheless. Fratricide-induced signaling has been well characterized for the CYAD-01 CAR and often compromised clinical manufacture.[16] We observed similar donor-dependent issues with expansion of CYAD-01 analog (*NKG2D-CD3ζ*) CAR T cells. Our data implicate the presence of an intact CD3ζ CAR endodomain accompanied by increased NKG2DL expression and CAR T cell activation in this finding and indicate that incorporation of a single ITAM-containing activation module can significantly increase cell yield. Consequently, the introduction of inactivating mutations within ITAMs 2 and 3 of the CYAD-01 CAR could potentially alleviate this problem.

A similar issue was reported during the pre-clinical development of a Lym-1-specific CAR that contained a fused 4-1BB + CD3ζ endodomain.[17] Ligand-dependent CAR signaling was triggered by low-level Lym-1 expression on the T cells. This issue was ameliorated by switching to a fused Dap10+Dap12 endodomain, reminiscent of the improved performance of our CARs that signal using this combination. In addition to containing a single ITAM, Dap12 contains an immunoreceptor tyrosine-based inhibitor motif,[36] which might mitigate the effects of ligand-dependent signaling via recruitment of Src homology 2-containing inositol phosphatase 1. Our expansion data generated using an extended panel of CARs suggest that delivery of this sub-optimal ligand-dependent signal is provided by an intact CD3ζ endodomain and is corrected by provision of a single ITAM-containing alternative.

It is increasingly appreciated that senescence is a key driver of CAR T cell failure.[27] We found that gene sets associated with cellular senescence were significantly reduced in activated *NKG2D/Dap10-12* adaptor CAR T cells, when compared to three CD3ζ-based CARs, including the CYAD-01 analog. In keeping with this, unstimulated *NKG2D/Dap10-12* T cells expressed significantly lower levels of the senescence markers CD57 and KLRG1,[27] while CD27 (which is lost in senescent T cells) was elevated when compared to control untransduced or NKG2D-engineered T cells. Moreover, gene sets involved in p53 signaling were downregulated in *NKG2D/Dap10-12* T cells. Notably p53 has also been implicated in cellular senescence[37] and is upregulated in CAR T cell products that have achieved poor results for patients.[38] Senescence leads to induction of cell surface NKG2DL expression on many cell types, rendering these cells

(B) Data shown in (A) but restricted to the four CAR T cell populations only.

(C) Volcano plot indicating differential gene expression when comparing stimulated *NKG2D/Dap10-12* T cells with all stimulated CD3ζ-containing CAR T cell populations. Significantly upregulated or downregulated genes (false discovery rate [FDR] < 0.05) are indicated in red.

(D) Gene set enrichment analysis (GSEA) running score plot of the ribosome gene signature (KEGG database) based on the comparison between *NKG2D/Dap10-12* T cells with CD3ζ-based CARs. The associated normalized enrichment score (NES) and FDR are reported.

(E) GSEA running score plot of expression of the oxidative phosphorylation pathway (KEGG database) based on the comparison between *NKG2D/Dap10-12* T cells with CD3ζ-based CARs. NES and FDR are reported.

(F) Heatmap of *Z* score transformed counts per millions of GSEA core enrichment genes of the ribosome signature (KEGG database) in *NKG2D/Dap10-12* T cells compared to CD3ζ-based CARs. Genes and samples are listed based on unsupervised hierarchical clustering of the underlying expression data.

(G) Heatmap of *Z* score transformed counts per millions of GSEA core enrichment genes of the oxidative phosphorylation pathway (KEGG database) in *NKG2D/Dap10-12* T cells compared to CD3ζ-based CARs. Genes and samples are listed based on unsupervised hierarchical clustering of the underlying expression data.

(H) Lollipop plot summarizing biologically relevant and statistically significant (FDR < 0.05) GSEA results based on the gene expression changes between *NKG2D/Dap10-12* and CD3ζ-based CAR T cells. Results for selected KEGG, REACTOME, and HALLMARK pathways are shown as circles, while position on the x axis indicates the NES. Circle color represents the associated FDR, and circle size corresponds to the overlap between the members of the signatures and the genes tested for differential expression in the current study.

susceptible to elimination by NKG2D-based CAR T cells.[39] In keeping with this, we observed increased NKG2DL expression on *NKG2D-CD3ζ* when compared to *NKG2D/Dap10-12* T cell cultures. These data strongly suggest that senescent NKG2DL^{+} CAR T cells are more efficiently removed during the expansion of *NKG2D/Dap10-12* compared to *NKG2D-CD3ζ* cultures, a point that would be expected to boost CAR T cell function.

Mitochondrial dysfunction is an established metabolic driver of senescence.[26,27] We found that *NKG2D/Dap10-12* CAR T cells had attributes indicative of sustained mitochondrial fitness. This may be the result of ligand-dependent co-stimulation that accompanies the preferential removal of senescent (i.e., NKG2DL^{+}) CAR T cells during their expansion, since Dap10 has also been reported to enhance the oxidative phosphorylation capacity of NK cells.[40] In agreement with this, pathway analysis consistently demonstrated elevated expression of gene sets associated with oxidative phosphorylation and respiratory electron transport in activated *NKG2D/Dap10-12* CAR T cells, when compared to CD3ζ-containing CAR T cells. GSEA also indicated that *NKG2D/Dap10-12* CAR T cells were transcriptionally programmed to increase ribosomal biogenesis and protein translation, attributes that place high bioenergetic demands on the cell but are strongly linked to enhanced T cell recall function.[41] This may be an important contributory factor to the sustained functional persistence of these CAR T cells *in vivo*.

Our RNA sequencing data also showed that several notable genes were upregulated in activated *NKG2D/Dap10-12* CAR T cells. First among these were *CXCL9* and *CXCL10*, both of which have been linked to enhanced CAR T cell anti-tumor activity.[42,43] Glutamine synthase contributes to T cell metabolism under conditions of activation, particularly in hypoxia, and was also transcriptionally elevated.[44] Expression of the *KCNMA1* potassium channel gene was also raised, a finding that may have significance since potassium channels have been implicated in T cell autoreactivity in rheumatoid arthritis.[45] By contrast, both *IGF1* and *IGF2* were downregulated, genes that are known to stimulate regulatory T cell proliferation.[46,47] *EMP1* was also downregulated and has been linked to impaired T cell anti-tumor activity in a genome-wide CRISPR screen.[48] *MAGI1* was also decreased and is a known negative regulator of mitogen-activated protein kinase/extracellular signal-regulated kinase (ERK) signaling.[49] Finally *KLRC1* was reduced in *NKG2D/Dap10-12* T cells, and increasing evidence implicates the encoded NKG2A gene product in the inhibitory regulation of T cell subsets.[50]

In conclusion, our data demonstrate the importance of careful optimization of CAR design when targeting NKG2DL. These data strongly support the clinical advancement of *NKG2D/Dap10-12* CAR T cell immunotherapy for the treatment of relapsed refractory NKG2DL-expressing solid tumors.

Limitations of the study

The use of i.p. and s.c. models to model tumor formation was criticized at peer review, prompting the consideration of alternative systems such as orthotopic models. Further investigation of DEGs and mechanisms underlying differences between NKG2D-based CARs is also warranted, examining which signaling motifs with the Dap10 and Dap12 molecules contribute to the enhanced mitochondrial fitness and anti-tumor activity of the *NKG2D/Dap10-12* CAR and how these impact on downstream signaling pathways. It would also be of interest to compare the provision of co-stimulation by Dap10 with other alternative candidates such as 4-1BB.

RESOURCE AVAILABILITY

Lead contact

Further information and requests for resources and reagents should be directed to and will be fulfilled by the lead contact, John Maher (john.maher@kcl.ac.uk).

Materials availability

Reagents generated in this study will be made available on request, but we may require a payment and/or a completed Materials Transfer Agreement if there is potential for commercial application.

Data and code availability

- Data: RNA sequencing data were deposited at NIH Gene Expression Omnibus (GEO) and are publicly available as of the date of publication at accession number GEO: GSE249511. All data reported in this paper will be shared by the lead contact upon request.
- Code: there was no new code developed as part of this study.
- All other items: any additional information required to re-analyze the data reported in this work paper is available from the lead contact upon request.

ACKNOWLEDGMENTS

This research was funded in part by the Wellcome Trust [J.O.; grant number 108874/B/15/Z]. For the purpose of open access, the author has applied a CC BY public copyright licence to any Author Accepted Manuscript version arising from this submission. Funding was also provided by Leucid Bio and the Experimental Cancer Medicine Centre at King's College London. K.R.F. was supported by the UK Medical Research Council (grant number MR/N013700/1) and King's College London member of the MRC Doctoral Training Partnership in Biomedical Sciences.

AUTHOR CONTRIBUTIONS

Conceptualization, J.M.; experimental work, J.O., D.L.-Y., M.G., F.K., C.M.H., G.T., K.R.F., P.D., R.M., C.B., C.T., and D.M.D.; bioinformatic analysis, J.O., R.E.B., D.C., and A.V.; methodology and data analysis, all authors; writing, J.M.; review and editing, all authors.

DECLARATION OF INTERESTS

J.M. is CSO, scientific founder, and shareholder of Leucid Bio. M.G., F.K., C.M.H., R.M., C.B., C.T., and D.M.D. are employees of Leucid Bio while P.D. is a former Leucid Bio employee. D.L.-Y. is undertaking a PhD studentship funded by Leucid Bio and has acted as a consultant for Leucid Bio. J.M., D.M.D., D.L.-Y., and F.K. are co-inventors on patent filings in relation to adaptor CAR technology.

STAR★METHODS

Detailed methods are provided in the online version of this paper and include the following:

- KEY RESOURCES TABLE
- EXPERIMENTAL MODEL AND STUDY PARTICIPANT DETAILS
  - Mice
  - Cell lines and tissue culture
  - Human study oversight

- METHOD DETAILS
  - Retroviral constructs
  - Transduction and expansion of human T-cells
  - Flow cytometry analysis
  - Total internal reflection fluorescence (TIRF) microscopy
  - Western Blotting
  - Polyfunctionality and activation/exhaustion marker analysis
  - Enzyme-linked Immunosorbent assay (ELISA)
  - Cytotoxicity assays
  - Tumor Re-stimulation assays
  - RNA analysis
  - Seahorse metabolic flux analysis
  - *In vivo* xenograft studies
- QUANTIFICATION AND STATISTICAL ANALYSIS

SUPPLEMENTAL INFORMATION

Supplemental information can be found online at https://doi.org/10.1016/j.xcrm.2024.101827.

Received: December 9, 2023
Revised: September 3, 2024
Accepted: October 21, 2024
Published: November 19, 2024

REFERENCES

1. Tan, G., Spillane, K.M., and Maher, J. (2023). The Role and Regulation of the NKG2D/NKG2D Ligand System in Cancer. Biology *12*, 1079. https://doi.org/10.3390/biology12081079.
2. Sheppard, S., Guedes, J., Mroz, A., Zavitsanou, A.M., Kudo, H., Rothery, S.M., Angelopoulos, P., Goldin, R., and Guerra, N. (2017). The immunoreceptor NKG2D promotes tumour growth in a model of hepatocellular carcinoma. Nat. Commun. *8*, 13930. https://doi.org/10.1038/ncomms13930.
3. Guerra, N., Tan, Y.X., Joncker, N.T., Choy, A., Gallardo, F., Xiong, N., Knoblaugh, S., Cado, D., Greenberg, N.M., and Raulet, D.H. (2008). NKG2D-deficient mice are defective in tumor surveillance in models of spontaneous malignancy. Immunity *28*, 571–580. https://doi.org/10.1016/j.immuni.2008.02.016.
4. Adami, A., and Maher, J. (2021). An overview of CAR T-cell clinical trial activity to 2021. Immunother. Adv. *1*, ltab004. https://doi.org/10.1093/immadv/ltab004.
5. Zhang, T., Lemoi, B.A., and Sentman, C.L. (2005). Chimeric NK-receptor-bearing T cells mediate antitumor immunotherapy. Blood *106*, 1544–1551. https://doi.org/10.1182/blood-2004-11-4365.
6. Ng, Y.Y., Tay, J.C.K., Li, Z., Wang, J., Zhu, J., and Wang, S. (2021). T Cells Expressing NKG2D CAR with a DAP12 Signaling Domain Stimulate Lower Cytokine Production While Effective in Tumor Eradication. Mol. Ther. *29*, 75–85. https://doi.org/10.1016/j.ymthe.2020.08.016.
7. Ang, W.X., Ng, Y.Y., Xiao, L., Chen, C., Li, Z., Chi, Z., Tay, J.C.K., Tan, W.K., Zeng, J., Toh, H.C., and Wang, S. (2020). Electroporation of NKG2D RNA CAR improves Vγ9Vδ2 T cell responses against human solid tumor xenografts. Mol. Ther. Oncolytics *17*, 421–430. https://doi.org/10.1016/j.omto.2020.04.013.
8. Lehner, M., Götz, G., Proff, J., Schaft, N., Dörrie, J., Full, F., Ensser, A., Muller, Y.A., Cerwenka, A., Abken, H., et al. (2012). Redirecting T cells to Ewing's sarcoma family of tumors by a chimeric NKG2D receptor expressed by lentiviral transduction or mRNA transfection. PLoS One *7*, e31210. https://doi.org/10.1371/journal.pone.0031210.
9. Fernández, L., Metais, J.Y., Escudero, A., Vela, M., Valentin, J., Vallcorba, I., Leivas, A., Torres, J., Valeri, A., Patino-Garcia, A., et al. (2017). Memory T Cells Expressing an NKG2D-CAR Efficiently Target Osteosarcoma Cells. Clin. Cancer Res. *23*, 5824–5835. https://doi.org/10.1158/1078-0432.CCR-17-0075.
10. Han, Y., Xie, W., Song, D.G., and Powell, D.J., Jr. (2018). Control of triple-negative breast cancer using ex vivo self-enriched, costimulated NKG2D CAR T cells. J. Hematol. Oncol. *11*, 92. https://doi.org/10.1186/s13045-018-0635-z.
11. Chang, Y.H., Connolly, J., Shimasaki, N., Mimura, K., Kono, K., and Campana, D. (2013). A chimeric receptor with NKG2D specificity enhances natural killer cell activation and killing of tumor cells. Cancer Res. *73*, 1777–1786. https://doi.org/10.1158/0008-5472.CAN-12-3558.
12. Song, D.G., Ye, Q., Santoro, S., Fang, C., Best, A., and Powell, D.J., Jr. (2013). Chimeric NKG2D CAR-expressing T cell-mediated attack of human ovarian cancer is enhanced by histone deacetylase inhibition. Hum. Gene Ther. *24*, 295–305. https://doi.org/10.1089/hum.2012.143.
13. Baumeister, S.H., Murad, J., Werner, L., Daley, H., Trebeden-Negre, H., Gicobi, J.K., Schmucker, A., Reder, J., Sentman, C.L., Gilham, D.E., et al. (2019). Phase I Trial of Autologous CAR T Cells Targeting NKG2D Ligands in Patients with AML/MDS and Multiple Myeloma. Cancer Immunol. Res. *7*, 100–112. https://doi.org/10.1158/2326-6066.CIR-18-0307.
14. Sallman, D.A., Kerre, T., Havelange, V., Poiré, X., Lewalle, P., Wang, E.S., Brayer, J.B., Davila, M.L., Moors, I., Machiels, J.P., et al. (2023). CYAD-01, an autologous NKG2D-based CAR T-cell therapy, in relapsed or refractory acute myeloid leukaemia and myelodysplastic syndromes or multiple myeloma (THINK): haematological cohorts of the dose escalation segment of a phase 1 trial. Lancet. Haematol. *10*, e191–e202. https://doi.org/10.1016/S2352-3026(22)00378-7.
15. Hendlisz, A., Rottey, S., Opyrchal, M., Odunsi, K., Machiels, J.-P., Saheb-jam, S., Shaza, L., Aspeslagh, S., Van den Eynde, M., Canon, J.-L., et al. (2018). Results and perspectives from Phase 1 studies assessing the safety and clinical activity of multiple doses of a NKG2D-based CAR-T therapy, CYAD-01, in metastatic solid tumors. Journal for Immunotherapy of Cancer *6*, P255.
16. Breman, E., Demoulin, B., Agaugué, S., Mauën, S., Michaux, A., Springuel, L., Houssa, J., Huberty, F., Jacques-Hespel, C., Marchand, C., et al. (2018). Overcoming Target Driven Fratricide for T Cell Therapy. Front. Immunol. *9*, 2940. https://doi.org/10.3389/fimmu.2018.02940.
17. Zheng, L., Ren, L., Kouhi, A., Khawli, L.A., Hu, P., Kaslow, H.R., and Epstein, A.L. (2020). A Humanized Lym-1 CAR with Novel DAP10/DAP12 Signaling Domains Demonstrates Reduced Tonic Signaling and Increased Antitumor Activity in B-Cell Lymphoma Models. Clin. Cancer Res. *26*, 3694–3706. https://doi.org/10.1158/1078-0432.CCR-19-3417.
18. Sigalov, A.B., and Uversky, V.N. (2011). Differential occurrence of protein intrinsic disorder in the cytoplasmic signaling domains of cell receptors. Self. Nonself. *2*, 55–72. https://doi.org/10.4161/self.2.1.14790.
19. Wang, E., Wang, L.C., Tsai, C.Y., Bhoj, V., Gershenson, Z., Moon, E., Newick, K., Sun, J., Lo, A., Baradet, T., et al. (2015). Generation of Potent T-cell Immunotherapy for Cancer Using DAP12-Based, Multichain, Chimeric Immunoreceptors. Cancer Immunol. Res. *3*, 815–826. https://doi.org/10.1158/2326-6066.CIR-15-0054.
20. Park, Y.P., Choi, S.C., Kiesler, P., Gil-Krzewska, A., Borrego, F., Weck, J., Krzewski, K., and Coligan, J.E. (2011). Complex regulation of human NKG2D-DAP10 cell surface expression: opposing roles of the γc cytokines and TGF-β1. Blood *118*, 3019–3027. https://doi.org/10.1182/blood-2011-04-346825.
21. Li, S., Zhao, R., Zheng, D., Qin, L., Cui, Y., Li, Y., Jiang, Z., Zhong, M., Shi, J., Li, M., et al. (2022). DAP10 integration in CAR-T cells enhances the killing of heterogeneous tumors by harnessing endogenous NKG2D. Mol. Ther. Oncolytics *26*, 15–26. https://doi.org/10.1016/j.omto.2022.06.003.
22. Groh, V., Wu, J., Yee, C., and Spies, T. (2002). Tumour-derived soluble MIC ligands impair expression of NKG2D and T-cell activation. Nature *419*, 734–738. https://doi.org/10.1038/nature01112.
23. Li, K., Mandai, M., Hamanishi, J., Matsumura, N., Suzuki, A., Yagi, H., Yamaguchi, K., Baba, T., Fujii, S., and Konishi, I. (2009). Clinical significance of the NKG2D ligands, MICA/B and ULBP2 in ovarian cancer: high

expression of ULBP2 is an indicator of poor prognosis. Cancer Immunol. Immunother. *58*, 641–652. https://doi.org/10.1007/s00262-008-0585-3.

24. Vyas, M., Reinartz, S., Hoffmann, N., Reiners, K.S., Lieber, S., Jansen, J.M., Wagner, U., Müller, R., and von Strandmann, E.P. (2017). Soluble NKG2D ligands in the ovarian cancer microenvironment are associated with an adverse clinical outcome and decreased memory effector T cells independent of NKG2D downregulation. OncoImmunology *6*, e1339854. https://doi.org/10.1080/2162402X.2017.1339854.

25. Lonez, C., Verma, B., Hendlisz, A., Aftimos, P., Awada, A., Van Den Neste, E., Catala, G., Machiels, J.P.H., Piette, F., Brayer, J.B., et al. (2017). Study protocol for THINK: a multinational open-label phase I study to assess the safety and clinical activity of multiple administrations of NKR-2 in patients with different metastatic tumour types. BMJ Open *7*, e017075. https://doi.org/10.1136/bmjopen-2017-017075.

26. Muliaditan, T., Halim, L., Whilding, L.M., Draper, B., Achkova, D.Y., Kausar, F., Glover, M., Bechman, N., Arulappu, A., Sanchez, J., et al. (2021). Synergistic T cell signaling by 41BB and CD28 is optimally achieved by membrane proximal positioning within parallel chimeric antigen receptors. Cell Rep. Med. *2*, 100457. https://doi.org/10.1016/j.xcrm.2021.100457.

27. Noll, J.H., Levine, B.L., June, C.H., and Fraietta, J.A. (2023). Beyond youth: Understanding CAR T cell fitness in the context of immunological aging. Semin. Immunol. *70*, 101840. https://doi.org/10.1016/j.smim.2023.101840.

28. Koneru, M., Purdon, T.J., Spriggs, D., Koneru, S., and Brentjens, R.J. (2015). IL-12 secreting tumor-targeted chimeric antigen receptor T cells eradicate ovarian tumors in vivo. OncoImmunology *4*, e994446. https://doi.org/10.4161/2162402X.2014.994446.

29. Vardhana, S.A., Hwee, M.A., Berisa, M., Wells, D.K., Yost, K.E., King, B., Smith, M., Herrera, P.S., Chang, H.Y., Satpathy, A.T., et al. (2020). Impaired mitochondrial oxidative phosphorylation limits the self-renewal of T cells exposed to persistent antigen. Nat. Immunol. *21*, 1022–1033. https://doi.org/10.1038/s41590-020-0725-2.

30. Maher, J., Brentjens, R.J., Gunset, G., Rivière, I., and Sadelain, M. (2002). Human T-lymphocyte cytotoxicity and proliferation directed by a single chimeric TCRzeta/CD28 receptor. Nat. Biotechnol. *20*, 70–75. https://doi.org/10.1038/nbt0102-70.

31. Zhao, Y., Wang, Q.J., Yang, S., Kochenderfer, J.N., Zheng, Z., Zhong, X., Sadelain, M., Eshhar, Z., Rosenberg, S.A., and Morgan, R.A. (2009). A herceptin-based chimeric antigen receptor with modified signaling domains leads to enhanced survival of transduced T lymphocytes and antitumor activity. J. Immunol. *183*, 5563–5574. https://doi.org/10.4049/jimmunol.0900447.

32. Feucht, J., Sun, J., Eyquem, J., Ho, Y.J., Zhao, Z., Leibold, J., Dobrin, A., Cabriolu, A., Hamieh, M., and Sadelain, M. (2019). Calibration of CAR activation potential directs alternative T cell fates and therapeutic potency. Nat. Med. *25*, 82–88. https://doi.org/10.1038/s41591-018-0290-5.

33. Obajdin, J., Davies, D.M., and Maher, J. (2020). Engineering of chimeric natural killer cell receptors to develop precision adoptive immunotherapies for cancer. Clin. Exp. Immunol. *202*, 11–27. https://doi.org/10.1111/cei.13478.

34. Guedan, S., Madar, A., Casado-Medrano, V., Shaw, C., Wing, A., Liu, F., Young, R.M., June, C.H., and Posey, A.D., Jr. (2020). Single residue in CD28-costimulated CAR-T cells limits long-term persistence and antitumor durability. J. Clin. Invest. *130*, 3087–3097. https://doi.org/10.1172/JCI133215.

35. Ajina, A., and Maher, J. (2018). Strategies to Address Chimeric Antigen Receptor Tonic Signaling. Mol. Cancer Therapeut. *17*, 1795–1815. https://doi.org/10.1158/1535-7163.MCT-17-1097.

36. Barrow, A.D., and Trowsdale, J. (2006). You say ITAM and I say ITIM, let's call the whole thing off: the ambiguity of immunoreceptor signalling. Eur. J. Immunol. *36*, 1646–1653. https://doi.org/10.1002/eji.200636195.

37. Rodier, F., and Campisi, J. (2011). Four faces of cellular senescence. J. Cell Biol. *192*, 547–556. https://doi.org/10.1083/jcb.201009094.

38. Kirouac, D.C., Zmurchok, C., Deyati, A., Sicherman, J., Bond, C., and Zandstra, P.W. (2023). Deconvolution of clinical variance in CAR-T cell pharmacology and response. Nat. Biotechnol. *41*, 1606–1617. https://doi.org/10.1038/s41587-023-01687-x.

39. Yang, D., Sun, B., Li, S., Wei, W., Liu, X., Cui, X., Zhang, X., Liu, N., Yan, L., Deng, Y., and Zhao, X. (2023). NKG2D-CAR T cells eliminate senescent cells in aged mice and nonhuman primates. Sci. Transl. Med. *15*, eadd1951. https://doi.org/10.1126/scitranslmed.add1951.

40. Basar, R., Daher, M.D., Uprety, N., Ensley, E., Nunez Cortes, A.K., Acharya, S., Shrestha, R., Liu, B., Shanley, M., Li, Y., et al. (2023). DAP10 Co-Stimulation Imparts Memory-like Features to CD5 Targeting Cord Blood Derived CAR-NK Cells. Blood *142*, 2089. https://doi.org/10.1182/blood-2023-187665.

41. Claiborne, M.D., Sengupta, S., Zhao, L., Arwood, M.L., Sun, I.M., Wen, J., Thompson, E.A., Mitchell-Flack, M., Laiho, M., and Powell, J.D. (2022). Persistent CAD activity in memory CD8+ T cells supports rRNA synthesis and ribosomal biogenesis required at rechallenge. Sci. Immunol. *7*, eabh4271. https://doi.org/10.1126/sciimmunol.abh4271.

42. Liu, T., Long, X., Zhang, Y., Jin, J., Chen, L., and Liu, A. (2022). IP-10 enhances the amplification capacity and antitumor activity of CAR-T cells in vitro and could influence positive outcomes in MM patients treated with CAR-T cell therapy. Int. Immunopharm. *112*, 109253. https://doi.org/10.1016/j.intimp.2022.109253.

43. Tian, Y., Wen, C., Zhang, Z., Liu, Y., Li, F., Zhao, Q., Yao, C., Ni, K., Yang, S., and Zhang, Y. (2022). CXCL9-modified CAR T cells improve immune cell infiltration and antitumor efficacy. Cancer Immunol. Immunother. *71*, 2663–2675. https://doi.org/10.1007/s00262-022-03193-6.

44. Wik, J.A., Chowdhury, A., Kolan, S., Bastani, N.E., Li, G., Alam, K., Grimolizzi, F., and Skålhegg, B.S. (2022). Endogenous glutamine is rate-limiting for anti-CD3 and anti-CD28 induced CD4+ T-cell proliferation and glycolytic activity under hypoxia and normoxia. Biochem. J. *479*, 1221–1235. https://doi.org/10.1042/BCJ20220144.

45. Tanner, M.R., Pennington, M.W., Chauhan, S.S., Laragione, T., Gulko, P.S., and Beeton, C. (2019). KCa1.1 and Kv1.3 channels regulate the interactions between fibroblast-like synoviocytes and T lymphocytes during rheumatoid arthritis. Arthritis Res. Ther. *21*, 6. https://doi.org/10.1186/s13075-018-1783-9.

46. Bilbao, D., Luciani, L., Johannesson, B., Piszczek, A., and Rosenthal, N. (2014). Insulin-like growth factor-1 stimulates regulatory T cells and suppresses autoimmune disease. EMBO Mol. Med. *6*, 1423–1435. https://doi.org/10.15252/emmm.201303376.

47. Yang, G., Geng, X.R., Song, J.P., Wu, Y., Yan, H., Zhan, Z., Yang, L., He, W., Liu, Z.Q., Qiu, S., et al. (2014). Insulin-like growth factor 2 enhances regulatory T-cell functions and suppresses food allergy in an experimental model. J. Allergy Clin. Immunol. *133*, 1702–1708.e5. https://doi.org/10.1016/j.jaci.2014.02.019.

48. Ye, L., Park, J.J., Dong, M.B., Yang, Q., Chow, R.D., Peng, L., Du, Y., Guo, J., Dai, X., Wang, G., et al. (2019). In vivo CRISPR screening in CD8 T cells with AAV-Sleeping Beauty hybrid vectors identifies membrane targets for improving immunotherapy for glioblastoma. Nat. Biotechnol. *37*, 1302–1313. https://doi.org/10.1038/s41587-019-0246-4.

49. Wildner, G., and Kaufmann, U. (2013). What causes relapses of autoimmune diseases? The etiological role of autoreactive T cells. Autoimmun. Rev. *12*, 1070–1075. https://doi.org/10.1016/j.autrev.2013.04.001.

50. van Montfoort, N., Borst, L., Korrer, M.J., Sluijter, M., Marijt, K.A., Santegoets, S.J., van Ham, V.J., Ehsan, I., Charoentong, P., André, P., et al. (2018). NKG2A Blockade Potentiates CD8 T Cell Immunity Induced by Cancer Vaccines. Cell *175*, 1744–1755.e15. https://doi.org/10.1016/j.cell.2018.10.028.

51. Lamprecht, B., Walter, K., Kreher, S., Kumar, R., Hummel, M., Lenze, D., Köchert, K., Bouhlel, M.A., Richter, J., Soler, E., et al. (2010). Derepression of an endogenous long terminal repeat activates the CSF1R proto-oncogene in human lymphoma. Nat Med. *16*, 571–579. https://doi.org/10.1038/nm.2129.

CellPress
OPEN ACCESS

52. Benson, G. (1999). Tandem repeats finder: a program to analyze DNA sequences. Nucleic Acids Res. *27*, 573–580. https://doi.org/10.1093/nar/27.2.573.

53. Roederer, M., Nozzi, J.L., and Nason, M.C. (2011). SPICE: exploration and analysis of post-cytometric complex multivariate datasets. Cytometry A. *79*, 167–174. https://doi.org/10.1002/cyto.a.21015.

54. Patro, R., Duggal, G., Love, M.I., Irizarry, R.A., and Kingsford, C. (2017). Salmon provides fast and bias-aware quantification of transcript expression. Nat. Methods *14*, 417–419. https://doi.org/10.1038/nmeth.4197.

55. Bolger, A.M., Lohse, M., and Usadel, B. (2014). Trimmomatic: a flexible trimmer for Illumina sequence data. Bioinformatics. *30*, 2114–2120. https://doi.org/10.1093/bioinformatics/btu170.

56. Kim, D., Langmead, B., and Salzberg, S.L. (2015). HISAT: a fast spliced aligner with low memory requirements. Nat Methods. *12*, 357–360. https://doi.org/10.1038/nmeth.3317.

57. Anders, S., Pyl, P.T., and Huber, W. (2015). HTSeq–a Python framework to work with high-throughput sequencing data. Bioinformatics. *31*, 166–169. https://doi.org/10.1093/bioinformatics/btu638.

58. Love, M.I., Huber, W., and Anders, S. (2014). Moderated estimation of fold change and dispersion for RNA-seq data with DESeq2. Genome Biol. *15*, 550. https://doi.org/10.1186/s13059-014-0550-8.

59. Martin, F.J., Amode, M.R., Aneja, A., Austine-Orimoloye, O., Azov, A.G., Barnes, I., Becker, A., Bennett, R., Berry, A., and Bhai, J. (2023). Ensembl 2023. Nucleic Acids Res. *6*, D933–D941. https://doi.org/10.1093/nar/gkac958.

60. Liberzon, A., Birger, C., Thorvaldsdóttir, H., Ghandi, M., Mesirov, J.P., and Tamayo, P. (2015). The Molecular Signatures Database (MSigDB) hallmark gene set collection. Cell Syst. *1*, 417–425. https://doi.org/10.1016/j.cels.2015.12.004.

61. Subramanian, A., Tamayo, P., Mootha, V.K., Mukherjee, S., Ebert, B.L., Gillette, M.A., Paulovich, A., Pomeroy, S.L., Golub, T.R., Lander, E.S., and Mesirov, J.P. (2005). Gene set enrichment analysis: a knowledge-based approach for interpreting genome-wide expression profiles. Proc. Natl. Acad. Sci. USA *102*, 15545–15550. https://doi.org/10.1073/pnas.0506580102.

62. Metsalu, T., and Vilo, J. (2015). ClustVis: a web tool for visualizing clustering of multivariate data using Principal Component Analysis and heatmap. Nucleic Acids Res. *43*, W566–W570. https://doi.org/10.1093/nar/gkv468.

63. Whilding, L.M., Parente-Pereira, A.C., Zabinski, T., Davies, D.M., Petrovic, R.M.G., Kao, Y.V., Saxena, S.A., Romain, A., Costa-Guerra, J.A., Violette, S., et al. (2017). Targeting of aberrant αvβ6 integrin expression in solid tumors using chimeric antigen receptor-engineered T cells. Mol. Ther. *25*, 259–273. https://doi.org/10.1016/j.ymthe.2016.10.012.

64. Davies, D.M., Foster, J., Van Der Stegen, S.J.C., Parente-Pereira, A.C., Chiapero-Stanke, L., Delinassios, G.J., Burbridge, S.E., Kao, V., Liu, Z., Bosshard-Carter, L., et al. (2012). Flexible targeting of ErbB dimers that drive tumorigenesis by using genetically engineered T cells. Mol. Med. *18*, 565–576. https://doi.org/10.2119/molmed.2011.00493.

65. Whilding, L.M., Halim, L., Draper, B., Parente-Pereira, A.C., Zabinski, T., Davies, D.M., and Maher, J. (2019). CAR T-cells targeting the integrin αvβ6 and co-expressing the chemokine receptor CXCR2 demonstrate enhanced homing and efficacy against several solid malignancies. Cancers *11*, 674. https://doi.org/10.3390/cancers11050674.

66. Love, M.I., Soneson, C., Hickey, P.F., Johnson, L.K., Pierce, N.T., Shepherd, L., Morgan, M., and Patro, R. (2020). Tximeta: Reference sequence checksums for provenance identification in RNA-seq. PLoS Comput. Biol. *16*, e1007664. https://doi.org/10.1371/journal.pcbi.1007664.

67. Hochberg, Y., and Benjamini, Y. (1990). More powerful procedures for multiple significance testing. Stat. Med. *9*, 811–818. https://doi.org/10.1002/sim.4780090710.

68. Liberzon, A., Subramanian, A., Pinchback, R., Thorvaldsdóttir, H., Tamayo, P., and Mesirov, J.P. (2011). Molecular signatures database (MSigDB) 3.0. Bioinformatics *27*, 1739–1740. https://doi.org/10.1093/bioinformatics/btr260.

69. Wickham, H. (2016). ggplot2: Elegant Graphics for Data Analysis, 1st edition (Springer), pp. 160–167. https://doi.org/10.1007/978-0-387-98141-3.

STAR★METHODS

KEY RESOURCES TABLE

REAGENT or RESOURCE	SOURCE	IDENTIFIER
Antibodies		
Anti-human CD3 APC/Cy7	BioLegend	Cat# 344818, RRID:AB_10645474
Anti-human CD3 BV605	BioLegend	Cat# 317322, RRID:AB_11126166
Anti-human CD3 (purified) (OKT3)	Miltenyi Biotec	Cat# 170-076-124, RRID:AB_2904535
Anti-human CD4 APC	BioLegend	Cat# 317416<br>RRID: AB_571944
Anti-human CD4 PE/Cy7 (OKT4)	BioLegend	Cat# 317414<br>RRID: AB_571959
Anti-human CD4 BV510 (SK3)	BioLegend	Cat# 344633<br>RRID: AB_2566016
Anti-human CD8 - PE-Cy7 (SK1)	BioLegend	Cat# 344711, RRID:AB_2044007
Anti-human CD8a Alexa Fluor 700 (OKT8)	eBioscience	Cat# 56-0086-82<br>RRID: AB_657756
Anti-human CD27 FITC (O323)	BioLegend	Cat# 302806<br>RRID: AB_314298
Anti-human CD27 BV605	BioLegend	Cat# 302830<br>RRID: AB_11204431
Anti-human CD45 APC	BioLegend	Cat# 304012, RRID:AB_314399
Anti-human CD45RO - PerCP/Cy5.5 (UCHL1)	BioLegend	Cat# 304221, RRID:AB_1575041
Anti-human CD57 - APC (QA17A04)	BioLegend	Cat# 393305, RRID:AB_2734459
Anti-human CD69 BV605 (FN50)	BioLegend	Cat# 310938<br>RRID: AB_2562307
Anti-human CD71 PerCP/Cy5.5 (CY1G4)	BioLegend	Cat# 334114<br>RRID: AB_2563175
Anti-human CD197 (CCR7) - APC (G043H7)	BioLegend	Cat# 353213, RRID:AB_353213
Anti-human CD223 (LAG-3) Alexa Fluor 647	BioLegend	Cat# 369304, RRID:AB_2566480
Anti-human CD223 (LAG-3) BV 785	BioLegend	Cat# 369321, RRID:AB_2716127
Anti-human CD279 (PD1) – APC/Cy7 (EH12.2H7)	BioLegend	Cat# 329921, RRID:AB_10900982
Anti-human CD279 (PD1) - PE/Dazzle 594 (EH12.2H7)	BioLegend	Cat# 329939, RRID:AB_2563658
Anti-human CD314 (NKG2D) - PE	Miltenyi Biotec	Cat# 130-111-645, RRID:AB_2657364
Anti-human CD314 (NKG2D) - PE	BioLegend	Cat# 320806, RRID:AB_492960
Anti-human CD314 (NKG2D) - PE/Cy7	BioLegend	Cat# 320812, RRID:AB_2234394
Anti-human CD366 (Tim-3) - APC	Biolegend	Cat# 345011, RRID:AB_2561717
Anti-human KLRG1 PE-Vio® 615 (REA261)	Miltenyi Biotec.	Cat# 130-120-427<br>RRID: AB_2733432
Anti-human Dap10 Alexa Fluor® 488	Bio-Techne	Cat# FAB9786G
Anti-human Dap12 Alexa Fluor® 647	BD Biosciences	Cat# 566603<br>RRID: AB_2869791
Anti-human MICA/B - APC	Bio-Techne	Cat# FAB13001A<br>RRID: AB_663946
Anti-human MICA/B - PE	Bio-Techne	Cat# FAB13001P<br>RRID: AB_663947
Anti-human ULBP1 - APC	Bio-Techne	Cat# FAB1380A<br>RRID: AB_2923476
Anti-human ULBP1 - PE	Bio-Techne	Cat# FAB1380P<br>RRID: AB_2687471

(*Continued on next page*)

Continued

REAGENT or RESOURCE	SOURCE	IDENTIFIER
Anti-human ULBP2/5/6 - APC	Bio-Techne	Cat# FAB1298A<br>RRID: AB_2257142
Anti-human ULBP2/5/6 - PE	Bio-Techne	Cat# FAB1298P<br>RRID: AB_2214693
Anti-human ULBP3 - APC	Bio-Techne	Cat# FAB1517A
Anti-human ULBP3 - PE	Bio-Techne	Cat# FAB1517<br>RRID: AB_10719122
Anti-human ULBP4 - APC	Bio-Techne	Cat# FAB6285A
Anti-human ULBP4 - PE	Bio-Techne	Cat# FAB6285P
Anti-human IL-2 - PE	BD Biosciences	Cat# 559334<br>RRID: AB_397231
Anti-human TNF-α - APC	BD Biosciences	Cat# 562084<br>RRID: AB_10893226
Anti-human IFN-γ - APC/Cy7	BioLegend	Cat# 502529<br>RRID: AB_10663411
Isotype Ctrl Antibody APC/Cyanine7 Mouse IgG1, κ	BioLegend	Cat# 400128<br>RRID: AB_2892538
Isotype Ctrl Antibody Brilliant Violet 605™ Mouse IgG2a, κ	BioLegend	Cat# 400270
Isotype Ctrl Antibody APC Mouse IgG2b, κ	BioLegend	Cat# 402206
Isotype Ctrl Antibody PE/Cyanine7 Mouse IgG2b, κ	BioLegend	Cat# 400326
Isotype Ctrl Antibody Brilliant Violet 510™ Mouse IgG1, κ	BioLegend	Cat# 400172
Isotype Ctrl Antibody PE/Cyanine7 Mouse IgG1, κ	BioLegend	Cat# 400126<br>RRID: AB_326448
Isotype Ctrl Antibody Mouse IgG2a kappa (eBM2a), Alexa Fluor™ 700	eBioscience	Cat# 56-4724-80<br>RRID: AB_494015
Isotype Ctrl (FC) Antibody FITC Mouse IgG1, κ	BioLegend	Cat# 400110<br>RRID: AB_2861401
Isotype Ctrl Antibody Brilliant Violet 605™ Mouse IgG1, κ	BioLegend	Cat# 400162
Isotype Ctrl (FC) Antibody APC Mouse IgG1, κ	BioLegend	Cat# 400122<br>RRID: AB_2665396
Isotype Ctrl Antibody PerCP/Cyanine5.5 Mouse IgG2a, κ	BioLegend	Cat# 400252<br>RRID: AB_10695169
Isotype Ctrl Antibody APC Mouse IgG2a, κ	BioLegend	Cat# 400220<br>RRID: AB_326468
Isotype Ctrl (FC) Antibody Alexa Fluor 647 Mouse IgG1, κ	BioLegend	Cat# 400130<br>RRID: AB_2800436
Isotype Ctrl Antibody Brilliant Violet 785™ Mouse IgG1, κ	BioLegend	Cat# 400170
Isotype Ctrl Antibody PE/Dazzle 594 Mouse IgG1, κ	BioLegend	Cat# 400176
Human TruStain FcX™ (Fc Receptor Blocking Solution)	BioLegend	Cat# 422302
IgG1 Antibody, anti-human, PE	Miltenyi Biotec.	Cat# 130-119-859
Mouse IgG2A APC-conjugated Antibody	Bio-Techne	Cat# IC003A
Mouse IgG2A PE-conjugated Antibody	Bio-Techne	Cat# IC003P
Mouse IgG2B APC-conjugated Antibody	Bio-Techne	Cat# IC0041A
Mouse IgG2B PE-conjugated Antibody	Bio-Techne	Cat# IC0041P
Live/dead nIR fixable viability dye	Thermo Fisher Scientific	Cat# L10119
NKG2D-Fc fusion protein	Acro Biosystems	Cat# NKD-H5265
OneComp eBeads	Invitrogen	Cat# 01-1111-41
Rabbit anti-NKG2D	Abcam	Cat# AB96606<br>RRID: AB_10676175
Goat anti-rabbit IgG HRP	Abcam	Cat# AB205718<br>RRID: AB_2819160

(*Continued on next page*)

REAGENT or RESOURCE	SOURCE	IDENTIFIER
Continued		
Anti-human Dap10 HRP	Santa Cruz Biotechnology	Cat# sc-133173 RRID: AB_2117803
Mouse anti-human Dap12 IgG2a	R&D Systems	Cat# MAB52401
Goat anti-mouse IgG2a HRP	Invitrogen	Cat# A10685 RRID: AB_2534065
OneComp eBeads	Invitrogen	Cat# 01-1111-41
Biological samples		
Peripheral blood mononuclear cells	Healthy donors	N/A
Chemicals, peptides, and recombinant proteins		
Cyto-Fast™ Fix/Perm Buffer Set	BioLegend	Cat# 426803
Hank's Balanced Salt Solution	Thermo Fisher Scientific	Cat# 14065049
Sodium Bicarbonate ($NaHCO_3$)	Fisher Scientific	Cat# BP328-500
Bovine Serum Albumin (BSA)	Thermofisher	Cat# BP1600-100
Sulfuric Acid (H_2SO_4)	Acros Organics	Cat# 424525001
Hydrogen peroxide (H_2O_2)	VWR	Cat# 23615.261
CellTrace™ CFSE Cell Proliferation Kit	Thermo Fisher Scientific	Cat# C34554
CellTrackerTM Deep Red	Thermo Fisher Scientific	Cat# C34565
D-luciferin	Bio-Techne	Cat# 122799
GeneJuice transfection reagent	Merck Chemicals Ltd	Cat# 70967-4
Live/Dead Fixable dead cell stain, blue	Thermo Fisher Scientific	Cat# L23105
Recombinant Human MICA-Fc Chimera Protein	Bio-Techne	Cat# 1300-MA-050
MTT (3-(4,5-Dimethylthiazol-2-yl)-2,5-diphenyl-2H-tetrazolium bromide)	Apollo Scientific	Cat# BID2165
Phytohemagglutinin-L	Sigma-Aldrich	Cat# 11249738001
Recombinant human IL-2	Peprotech EC	Cat# 200-02
Transforming growth factor β1	BioTechne	Cat# 240-B
RetroNectin	Takara Clontech	Cat# T100B
RIPA buffer	Merck	Cat# R0278-50ML
Pierce™ BCA Protein Assay Kits	Thermo Fisher Scientific	Cat# 23225
Pierce™ Dithiothreitol	Thermo Fisher Scientific	Cat# A39255
NuPAGE™ LDS Sample Buffer (4X)	Thermo Fisher Scientific	Cat# NP0007
NuPAGE™ Bis-Tris Midi Protein Gels, 4 to 12%	Thermo Fisher Scientific	Cat# WBT41212BOX
SeeBlue™ Plus2 Pre-stained Protein Standard	Thermo Fisher Scientific	Cat# LC5925
PageRuler™ Plus Prestained Protein Ladder, 10 to 250 kDa	Thermo Fisher Scientific	Cat# 26619
NuPAGE™ MES SDS Running Buffer (20X)	Thermo Fisher Scientific	Cat# NP0002
cOmplete protease inhibitors	Roche	Cat# 4693159001
0.45μM PVDF Membrane	Thermo Fisher Scientific	Cat# 88518
NuPAGE™ Transfer Buffer (20X)	Thermo Fisher Scientific	Cat# NP0006
Ponceau S staining solution	Thermo Fisher Scientific	Cat# A40000279
TRIS-buffered saline (TBS, 10X) pH 7.4, for Western blot	Thermo Fisher Scientific	Cat# J62938.K7
SuperSignal™ West Pico PLUS Chemiluminescent Substrate	Thermo Fisher Scientific	Cat# 34580
Phorbol 12-myristate 13-acetate	Merck	Cat# P8139
Ionomycin	Merck	Cat# I9657
Poly-L-lysine	Sigma-Aldrich	Cat# P4707
Zombie Violet	BioLegend	Cat# 423113
Critical commercial assays		
Apoptosis Kit	ThermoFisher	Cat# A10788
IFN-γ ELISA kit	ThermoFisher Scientific	Cat# 88-7316-76, RRID:AB_2575072

(*Continued on next page*)

Continued

REAGENT or RESOURCE	SOURCE	IDENTIFIER
IL-2 ELISA kit	ThermoFisher Scientific	Cat# 88-7025-76, RRID:AB_2574956
One-Step^{TM} Luciferase Assay System	BPS Bioscience	Cat# 60690-1
PlasmoTest^{TM}	InvivoGen	Cat# rep-pt1
STR typing	NorthGene	N/A
RNeasy® Mini Kit	Qiagen	Cat# 74104
Deposited data		
RNA-seq gene expression data	This paper	GEO: GSE249511
Experimental models: Cell lines		
293VEC-RD114^{TM}	Dr Manuel Caruso, Center de recherche du CHU de Québec, Canada	https://www.biovecpharma.com/products.php?id=19, accessed December 09,2023
HEK293T cells	European Collection of Authenticated Cell Cultures	Cat#12022001 RRID:CVCL_0063
PG13	European Collection of Cell Cultures (ECACC)	ATCC Cat# CRL-10685, RRID:CVCL_8933
BxPC3	European Collection of Cell Cultures (ECACC)	Cat# 93120816, RRID:CVCL_0186
PaTu-8902	Professor Claire Wells, King's College London	RRID:CVCL_1845
HN3	Ludwig Institute for Cancer Research, London, UK	RRID:CVCL_8126
SKOV3	PerkinElmer	Cat# BW119276 RRID CVCL_0532
Kuramochi	Japanese Collection of Research Bio-resources Cell Bank	Cat# JCRB0098 RRID CVCL_1345
A2780	European Collection of Cell Cultures (ECACC)	Cat# 93112519 RRID CVCL_0134
Ren	Prof D Fennell, University of Leicester, UK	RRID CVCL_M202
Ju77	European Collection of Cell Cultures (ECACC)	Cat# 10092309, RRID:CVCL_2536
H226	American Tissue Culture Collection (ATCC)	Cat# CRL-5826 RRID:CVCL_1544
Mesothelioma PDX_008	This paper	N/A
Experimental models: Organisms/strains		
Mouse: NSG® (NOD.Cg-Prkdc^{scid} Il2rg^{tm1Wjl}/SzJ)	Charles River	Strain code: 614
Recombinant DNA		
SFG *NKG2D/Dap10-12*	This manuscript	N/A
SFG NKG2D	This manuscript	N/A
SFG NKG2D/Dap10	This manuscript	N/A
SFG *NKG2D-CD3ζ*	This manuscript	N/A
SFG *NKG2D-CD3ζ/Dap10*	This manuscript	N/A
SFG *NKG2D/Dap10-CD3ζ*	This manuscript	N/A
SFG *NKG2D/Dap10-CD3ζ(1XX)*	This manuscript	N/A
SFG *NKG2D-Dap10-12*	This manuscript	N/A
SFG *N(Tr.)-CD3ζ*	This manuscript	N/A
SFG *N(Tr.)-CD3ζ*	This manuscript	N/A
SFG *NKG2D-Dap10-CD3ζ*	This manuscript	N/A
SFG *N(Tr.)-Dap10-CD3ζ*	This manuscript	N/A
SFG *NKG2D-Dap10-12/Dap10*	This manuscript	N/A
SFG *N(Tr.)-Dap10-12/Dap10*	This manuscript	N/A

(*Continued on next page*)

Continued

REAGENT or RESOURCE	SOURCE	IDENTIFIER
SFG *NKG2D/Dap10(Tr.)-CD28⁻CD3ζ*	This manuscript	N/A
RFP/ffLuc	This lab (Lamprecht et al., 2010[51])	Lamprecht et al., 2010[51]
RD114	Gift of Prof. M. Collins, University College London	N/A
pEQ-Pam3	Gift of Dr. M. Pulé, University College London	N/A
Software and algorithms		
FACSDiva	BD Biosciences	N/A
CytExpert	Beckman Coulter	N/A
FlowJo	FlowJo, LCC, BD Biosciences	N/A
Seahorse Analytics Program	Agilent Technologies	N/A
MARS Data Analysis Software	BMG Labtech	N/A
BMG Labtech Control Software	BMG Labtech	N/A
GraphPad Prism version 5.0, 6.0 and 7.0	GraphPad software	N/A
Excel for Mac 2011	Microsoft	N/A
Living Image Software	PerkinElmer	N/A
R (version 4.2.1)	R Core Team	N/A
Fiji	Fiji Downloads	N/A
Gene Designer	Atum Incorporated	N/A
Signal Predict	DTU Health Tech	N/A
Tandem Repeats Finder	Benson, 1999[52]	Benson, 1999[52]
Spice	Roederer et al., 2011[53]	Roederer et al., 2011[53]
Salmon (version 1.9.0)	Patro et al., 2017[54]	Patro et al., 2017[54]
R package tximeta	Love et al., 2019	PPR: PPR93747
R package trimmomatic	Bolger et al., 2014[55]	Bolger et al., 2014[55]
HISAT	Kim et al., 2015[56]	Kim et al., 2015[56]
HTSeq	Anders et al., 2015[57]	Anders et al., 2015[57]
DESeq2	Love et al., 2014[58]	Love et al., 2014[58]
Ensembl	Martin et al., 2023[59]	Martin et al., 2023[59]
MSigDB	Liberzon et al., 2015[60]	Liberzon et al., 2015[60]
GSEA	Subramanian et al., 2005[61]	Subramanian et al., 2005[61]
R package pheatmap	Raivo Kolge, 2019	N/A
R package ggplot2	Hadley Wickham, 2016	N/A
ClustVis	Metsalu et al., 2015[62]	Metsalu et al., 2015[62]
Other		
Antibiotic Antimycotic	Thermo Fisher Scientific	Cat# 15240096
DMEM	Lonza	Cat# BE12-709F
Glutamax	Thermo Fisher Scientific	Cat# 35050061
RPMI 1640 with L-Glutamine	Lonza	Cat# BE12-702F
IMDM	Thermo Fisher Scientific	Cat# 12440061
Dulbecco's Phosphate Buffered Saline	Merck	Cat# 8537
Human AB serum	Merck	Cat# H4522
XF RPMI Medium pH 7.4	Agilent Seahorse	Cat# 103576-100
XF 1.0 M Glucose solution	Agilent Seahorse	Cat# 103577-100
XF 100 mM Pyruvate solution	Agilent Seahorse	Cat# 103578-100
XF 200 mM Glutamine solution	Agilent Seahorse	Cat# 103579-100
XFe96/XF Pro PDL FluxPak Mini	Agilent Seahorse	Cat# 103798-100
Oligomycin A	Santa Cruz Biotechnology	Cat# 579-13-5

(*Continued on next page*)

Continued

REAGENT or RESOURCE	SOURCE	IDENTIFIER
BAM15	Cambridge Bioscience	Cat# CAY17811-1
Rotenone	Cambridge Bioscience	Cat# 13995-25mg-CAY
Antimycin A	Scientific Laboratory Supplies	Cat# A8674-50MG
Trypan Blue Solution (0.4%)	Thermo Fisher Scientific	Cat# 15250061
Lab-Tek eight well chamber	Thermo Fisher Scientific	Cat# 177402 PK

EXPERIMENTAL MODEL AND STUDY PARTICIPANT DETAILS

Mice

All *in vivo* experimentation adhered to U.K. Home Office guidelines, as specified in project licence number 70/7794 or P23115EBF and was approved by the King's College London animal welfare and ethical review body (AWERB). NSG (NOD.Cg-Prkdc^{scid} Il2rg^{tm1Wjl}/SzJ) mice were purchased from Charles River Laboratories and were 6–10 weeks old when used for experiments. Female mice were used for all ovarian cancer studies while male and female mice for all other xenograft studies. Mice were randomly allocated to experimental groups based on similar average tumor burden prior to treatment.

Mice were maintained in individually ventilated cages (IVCs) in Biological Services Units at King's College London. They were provided with an appropriate environment (e.g., nesting material, shelter, environmental enrichment etc.), including sufficient space and complexity to satisfy their normal behavior, in compliance with the standard code of practice required by the UK Animals (Scientific) Procedures Act 1986. Animals were maintained under barrier conditions, to minimize infection risk. Animals were not housed in isolation when possible. To minimize animal stress, tail marks using a pen were used to identify individual mice. Animals were handled using cleaned, gloved hands particularly when handling between cages to prevent possible transmission of infection. If adverse effects occurred (e.g., features of cytokine release syndrome), animals were subject to increased monitoring and consideration given to interventions that may reduce suffering (e.g., mushy diet).

Cell lines and tissue culture

Cell lines and their origin are listed in the key resources table. Tumor cell lines were grown in R10 or D10 medium, respectively comprising RPMI or DMEM supplemented with 10% FBS and GlutaMax. PG13 and 293VEC-RD114 cells retroviral packaging cells were maintained in D10. Human peripheral blood mononuclear cells and engineered T-cells were cultured in RPMI +5% human AB serum (R5 medium) containing IL-2 (100U/mL). Cells were maintained at 37°C in a humidified atmosphere of 5% CO_2. Cell lines were validated by STR typing and were routinely monitored for mycoplasma contamination. Where indicated, cell lines were engineered to express RFP/ffLuc by retroviral transduction, as described.[63]

Human study oversight

Following the provision of informed consent to a trained phlebotomist or clinician, blood samples were obtained from healthy male and female volunteers aged between 18 and 65 years old with approval of a National Health Service Research Ethics Committee (ref. 09/H0804/92, 18/WS/0047 and 22/LO/0357).

METHOD DETAILS

Retroviral constructs

All CARs were constructed by gene synthesis and cloning (Genscript, Hong Kong, China and Leiden, The Netherlands) using human codon-optimized sequences and ligation of digested DNA fragments as appropriate. Synthetic cDNA sequences were generated using Gene Designer. Predicted signal cleavage sites were checked using Signal Predict. Unwanted direct repeat sequences were identified using Tandem Repeats Finder. Gene expression was achieved using the SFG retroviral vector. Synthetic DNA sequences were flanked on the 5′ side by an NcoI restriction site (coincides with start codon in the SFG vector) and with a 3′ XhoI site downstream of the stop codon. Inserted human cDNA sequences consisted of the following elements (numbered to match Figure 5A).

(1) *NKG2D-Dap10-12*: start codon (ATG) – Dap12 endodomain (amino acids (aa) 62–113) – Dap10 endodomain (aa 70–93) – full length NKG2D. This arrangement was required since NKG2D is a type II transmembrane protein.
(2) *N(Tr.)-Dap10-12*: ATG – Dap12 endodomain – Dap10 endodomain – NKG2D extracellular and transmembrane domain (aa 52–216).
(3) *NKG2D-CD3ζ*: ATG – CD3ζ endodomain (aa 52–164) - full length NKG2D.
(4) *N(Tr.)-CD3ζ*: ATG – CD3ζ endodomain – NKG2D extracellular and transmembrane domain.
(5) *NKG2D-Dap10-CD3ζ*: ATG – CD3ζ endodomain – Dap10 endodomain – full length NKG2D.

(6) *N(Tr.)-Dap10-CD3ζ*: ATG – CD3ζ endodomain – Dap10 endodomain – NKG2D extracellular and transmembrane domain.
(7) *NKG2D/Dap10-12*: full length Dap10 – Dap12 endodomain - RRKR (furin cleavage site) – [serine-glycine (SG)]$_2$ linker - *Porcine Teschovirus* (P2A) ribosomal skip peptide – full length human NKG2D.
(8) *NKG2D-Dap10-12/Dap10*: full length Dap10 – RRKR – [SG]$_2$ – P2A – ATG – Dap12 endodomain – Dap10 endodomain – full length NKG2D.
(9) *N(Tr.)-Dap10-12/Dap10*: full length Dap10 – RRKR – [SG]$_2$ – P2A – ATG – Dap12 endodomain – Dap10 endodomain – NKG2D extracellular and transmembrane domain.
(10) *NKG2D-CD3ζ/Dap10*: full length DAP10 - RRKR – [SG]$_2$ - P2A - start codon (ATG) – CD3ζ endodomain – full length NKG2D.
(11) *NKG2D/Dap10-CD3ζ*: full length Dap10 - CD3ζ endodomain- RRKR – [SG]$_2$ - P2A – full length NKG2D.
(12) *NKG2D/Dap10-CD3ζ(1XX)*: generated by insertional mutagenesis, converting all four tyrosine codons in CD3ζ ITAMs 2 and 3 to phenylalanine codons.
(13) *NKG2D/Dap10(Tr.)-CD28$^-$CD3ζ*: Dap10 leader, extracellular and transmembrane domain (aa 1–69) – CD28 endodomain (aa 180–220) – CD3ζ endodomain – RRKR – [SG]$_2$ – P2A – full length NKG2D.

The NKG2D/Dap10 construct consisted of full length Dap10 - RRKR – [SG]$_2$ - P2A – NKG2D. The SFG A20-28z CAR (specific for αvβ6 integrin),[63] SFG RFP/ffLuc[63] and SFG T4[64] (encodes panErbB CAR) retroviral vectors have been described previously.

Transduction and expansion of human T-cells

Viral vector was prepared as described using PG13 cell lines, 293VEC-RD114 cells or by triple transfection of 293T cells. To achieve the latter, 1.65x10^6 low passage 293T cells in 11mL IMDM +10% FBS were evenly distributed in a 10cm plate. After 8-24h, GeneJuice (30μL) was added to 470μL IMDM (no serum) and mixed gently. After incubation for 5 min at room temperature, 3.125μg RD114 plasmid, 4.6875μg pEQ-Pam3 plasmid and 4.6875μg SFG vector of interest were added to the GeneJuice/medium mixture, mixed gently and incubated for 15 min at room temperature. The transfection mixture was dropwise to the plate and gently swirled to ensure even distribution. After incubation for 48 h at 37°C, 5% CO_2, medium was removed for snap freezing using an ethanol dry ice bath and replaced. After a further 24h, this procedure was repeated. Frozen virus was stored in aliquots at −80°C. Retroviral transduction and culture of phytohemagglutinin-activated T-cells using RetroNectin-coated plasticware was performed as described.[30,65]

Flow cytometry analysis

All cell staining reactions were performed on ice. For intracellular antigen detection, cells were stained with a fixable Live/Dead dye before being stained for surface proteins for 30 min on ice. Intracellular staining was performed by fixation with 0.01% formaldehyde followed by permeabilization using PBS +0.5% BSA +0.1% saponin. Cells were subsequently stained for intracellular proteins for 30 min at 4°C. All gates were set using isotype control antibodies or fluorescence minus one controls. Where necessary, a viability stain was included and non-specific binding of the antibodies was limited by using an appropriate Fc blocking reagent prior to the staining steps. Where indicated, to compare MFI across experiments, data were normalized against MFI of *NKG2D/Dap10-12* cells, which was set to 100 arbitrary units. To test for fratricide, *NKG2D/Dap10-12* CAR T-cells were labeled with CDTR (1μM) while *NKG2D/Dap10-12*, NKG2D or untransduced T-cells were labeled with CFSE (1μM). Cells were co-incubated for 48 h prior to analysis by flow cytometry. All flow cytometry was performed using a BD LSRFortessa cytometer with BD FACSDiva software or Cytoflex cytometer with CytExpert software and data were analyzed using FlowJo, LLC.

Total internal reflection fluorescence (TIRF) microscopy

Glass coverslips were etched with piranha (2:1 H_2SO_4:H_2O_2) solution for 12 min and subsequently washed with ultrapure water and ethanol before drying with argon. They were subsequently attached to a Lab-Tek 8-well chamber slide system. Approximately 3 x 10^5 cells in 150μL imaging buffer (0.1% BSA, HBSS and $NaHCO_3$) were loaded into chamber's well pre-coated with Poly-L-lysine. Five minutes after loading, cells were fixed by addition of 225μL Cyto-Fast Fix Perm Solution to each well and incubation for 20 min at room temperature. Cells were then washed into Cyto-Fast Perm Wash solution and subsequently blocked with 5% human AB serum in Cyto-Fast Perm Wash solution for 1 h before staining. Alexa Fluor 488 anti-human DAP10 antibody, Alexa Fluor 647 anti-human DAP12 antibody and PE anti-human NKG2D antibody (3μL each) were added into each sample. Cells were stained for 20 min in the dark at room temperature. Wells were then washed with 250μL of PBS. Images were collected using a Nikon Eclipse Ti-E TIRF microscope with 100x, 1.49 NA oil-immersion objective, 488-, 561- and 637-nm diode lasers (Coherent OBIS). Images were then processed and analyzed using Fiji software.

Western Blotting

293T cells were transfected with vector encoding plasmid alone as described in the Transduction section above. Five million cells were pelleted and washed twice in cold PBS. Cells were lysed in 1mL RIPA buffer with protease inhibitors and mixed for 15 min at 4°C. The lysis mixture was then centrifuged at 14,000g for 15 min at 4°C. The resulting protein supernatant was collected and transferred to a new tube. A Pierce Bicinchoninic acid (BCA) assay was performed to quantify protein concentration. 1x Lithium Dodecyl Sulfate (LDS) sample buffer and 50mM dithiothreitol were added to 30μg protein. Samples were heated at 95°C for 15 min and allowed to cool for 5 min. Samples were loaded onto a NuPAGE 4–12% Bis-Tris gel alongside SeeBlue Plus2 and PageRuler Plus

protein ladders. Electrophoresis was performed at 150V for ~45 min using 1X MES running buffer. Proteins were transferred to 0.45um polyvinylidene difluoride (PVDF) membrane at 20V for 1 h using 1x transfer buffer with 10% methanol. Protein transfer was confirmed using Ponceau S solution. Membranes were blocked in 5% non-fat milk in TBS overnight at 4°C. Membranes were stained with 1μg/mL primary antibodies for 2 h at room temperature. Membranes were washed 3 times in 0.1% Tween 20 PBS. Membranes were stained with 50 ng/mL secondary antibodies for 1 h at room temperature. Membranes were washed 6 times in 0.1%Tween 20 PBS. SuperSignal West Pico PLUS chemiluminescent substrate was added to membranes for 5 min. Membranes were imaged using a G:BOX Chemi XX6.

Polyfunctionality and activation/exhaustion marker analysis

CAR T-cells (1 x 10^6 cells/mL) were stimulated on immobilised OKT3 (800 ng/mL) or with PMA (50 ng/mL) and ionomycin (1μg/mL) for 24 h prior to flow cytometry analysis for the indicated cell surface or intracellular antigens. Data was analyzed using SPICE version 6.1.

Enzyme-linked Immunosorbent assay (ELISA)

Supernatants collected from co-cultures of tumor cells with CAR T-cells were analyzed using a human IFN-γ or human IL2 ELISA as described by the manufacturers, with a limit of sensitivity of 1 pg/mL.

Cytotoxicity assays

Tumor cells were incubated with T-cells at specified target to effector ratios. Residual tumor cell viability was quantified using an MTT or luciferase assay as follows. In the case of MTT assays, supernatant and residual T-cells were first removed and MTT was added at 500 μg/mL in D10 medium for 40 min at 37°C and 5% CO_2. Formazan crystals were resuspended in DMSO and absorbance was measured at 560 nm. In the case of luciferase assays, D-luciferin was added to the co-culture at 150 mg/mL immediately prior to luminescence reading. In each case, tumor cell viability was calculated as follows: $(\textit{absorbance or luminescence of tumor cells cultured with T} - \textit{cells}/\textit{absorbance or luminescence of untreated monolayer alone})\ x\ 100\%$.

Tumor Re-stimulation assays

Tumor cell lines were dissociated using trypsin and seeded at a density of 1 x 10^5 cells per well in a non-tissue culture-treated 24 well plate for 24h. Next, T-cells were added at a 1:1 CAR^+ T cell effector-to-target ratio. After each alternating 3–4 days re-stimulation cycle, tumor cell viability was assessed using MTT or luciferase assays, as described above. Re-stimulations were considered successful if <60% of tumor cells remained viable; otherwise the process was terminated. Upon completion of each re-stimulation cycle, T-cells were sedimented, re-suspended in R5 media, counted and added to a fresh tumor monolayer that had been prepared as above. This process was repeated upon completion of each re-stimulation cycle. Maximum fold expansion was determined as the ratio between the maximum cell count achieved over the duration of the re-stimulation assay compared to the starting cell number.

RNA analysis

Non-tissue culture-treated 24-well plates were coated with 0.1 μg/mL of human MICA-Fc fusion protein in PBS. The next day, excess PBS was removed and 2 x 10^5 of the indicated CAR T cell populations (n = 3 independent donors) were added per well in 1 mL of R5 medium. After 24h, T-cells were harvested into 15mL Falcon tubes and washed with 5 mL of PBS. Cells (1:2 dilution with Trypan Blue) were then counted using the LUNA-FL Dual Fluorescence Cell Counter (Logos Biosystems). A total of 1 × 10^6 T cells per condition were aliquoted and centrifuged at 800 g for 10 min. Pellets were stored at −20°C until shipment for RNA sequencing.

Nucleic acid extraction, sample QC, library preparation and sequencing were performed by Genewiz (Genomics from Azenta Life Sciences). Paired-end sequencing was performed on the Illumina NovaSeq, with a 2x150bp configuration and an estimated 20 million paired-end reads per sample.

FASTQ files were generated by Genewiz and initial sample QC was run, providing a report detailing RNA concentration as well as RNA quality numbers. Quality of the sequencing data was studied by the package fastqc (http://www.bioinformatics.babraham.ac.uk/projects/fastqc/, accessed August 1st, 2023). Transcript quantification was performed using Salmon[54] (v. 1.9.0) against a decoy-aware transcriptome generated from the GENCODE reference assembly (v. 41) of the human genome. Transcript-level data were imported into R (v. 4.2.1; https://www.r-project.org, accessed 01.08.2022) and gene-level count data were generated with the tximeta[66] package.

Data for Ensembl genes with no associated ENTREZ gene identifier were discarded; counts for Ensembl genes mapped to the same ENTREZ gene identifier were summed up in each sample. Differential expression analysis was performed in R using DESeq2[58] and adjusting for donor identity. Only genes with at least 0.5 counts per million in at least 2 samples and not coding for immunoglobulin light and heavy chains and known B-cell markers were tested. The Benjamini and Hochberg procedure[67] was applied for multiple testing correction.

Enrichment of signatures in MSigDB[60,68] was assessed using GSEA.[61] Genes were ranked by decreasing scores calculated by taking the geometric mean between the absolute value of the log fold change and the p-value from DEseq2 following log10

transformation and change of sign. The sign of the log fold change was finally multiplied to the ranking measure. These calculations were executed in R using the packages msigdbr and fsgsea with default parameters.

Heatmaps of *Z* score transformed counts per millions were generated using the pheatmap package (https://cran.r-project.org/web/packages/pheatmap/index.html, accessed March 1st, 2024). All plots were generated using the ggplot2 package.[69] Genes were labeled as differentially upregulated if the log2 fold change in their expression was >0.6, with an adjusted *p*-value <0.05. Conversely, genes were labeled as differentially downregulated if the log2 fold change in their expression was < -0.6, with an adjusted *p*-value <0.05.

Seahorse metabolic flux analysis

Real-time analysis of oxygen consumption rates (OCR) and extracellular-acidification rates (ECAR) of untransduced T-cells and CAR T-cells post-transduction and expansion for 10 days were assessed using a Seahorse XFe-96 analyser (Agilent Technologies). Cells were resuspended in Agilent Seahorse XF RPMI medium pH 7.4 supplemented with glucose (10mM), glutamine (2mM), and sodium pyruvate (1mM) and 1 x 10^5 cells/well were seeded in a Seahorse XFe96 Cell Culture PDL-coated Microplate (all Agilent Technologies). Measurements of ECAR and OCR were performed prior to and following the sequential addition of Oligomycin A (1.5μM), BAM15 (2.5μM) and Rotenone (0.5μM) plus Antimycin A (0.5μM). Data was analyzed using Seahorse Analytics (Agilent Technologies) to calculate basal OCR, basal ECAR and spare respiratory capacity (SRC).

In vivo xenograft studies

Tumor cells were inoculated i.p. or s.c. as specified in individual experiments. Once tumors were established, indicated by caliper measurements or bioluminescence imaging (BLI), T-cells were administered using the i.p. or i.v. routes at doses specified in individual experiments. BLI was performed using an IVIS Spectrum Imaging platform (PerkinElmer) with Living Image software. To monitor tumor status, mice were injected i.p. with D-luciferin (150 mg/kg) and imaged under isoflurane anesthesia after 20 min. In all experiments, animals were inspected daily and weighed weekly.

QUANTIFICATION AND STATISTICAL ANALYSIS

All data are derived from biological replicates involving independent donors unless otherwise indicated. For analysis of multiple groups, statistical analysis was performed using one-way or two-way ANOVA test, when there were one or two independent variables respectively, followed by Tukey's multiple comparisons test. For non-parametrically distributed data, a Kruskal Wallis or Friedman test was performed and correlation analysis determined using a Spearman test. Survival data were analyzed using a Log rank (Mantel-Cox) test. When only 2 groups were compared, a paired or unpaired Student's t test or Mann-Whitney test was performed, depending on normality of the data and whether data were dependent or independent. Survival was analyzed using a Log Rank (Mantel-Cox) test. All statistical analyses were performed using GraphPad Prism version 9.1. Significance is indicated as follows: ****$p < 0.0001$; ***$p < 0.001$; **$p < 0.01$ *$p < 0.05$.

Cell Reports Medicine, Volume *5*

Supplemental information

Solid tumor immunotherapy using NKG2D-based adaptor CAR T cells

Jana Obajdin, Daniel Larcombe-Young, Maya Glover, Fahima Kausar, Caroline M. Hull, Katie R. Flaherty, Ge Tan, Richard E. Beatson, Phoebe Dunbar, Roberta Mazza, Camilla Bove, Chelsea Taylor, Andrea Bille, Katelyn M. Spillane, Domenico Cozzetto, Alessandra Vigilante, Anna Schurich, David M. Davies, and John Maher

Supplemental Information

Solid tumor immunotherapy using NKG2D-based adaptor CAR

T-cells

Jana Obajdin, Daniel Larcombe-Young, Maya Glover, Fahima Kausar, Caroline M. Hull, Katie R. Flaherty, Ge Tan, Richard E. Beatson,, Phoebe Dunbar, Roberta Mazza, Camilla Bove, Chelsea Taylor, Andrea Bille, Katelyn M. Spillane, Domenico Cozzetto, Alessandra Vigilante, Anna Schurich, David M. Davies, John Maher

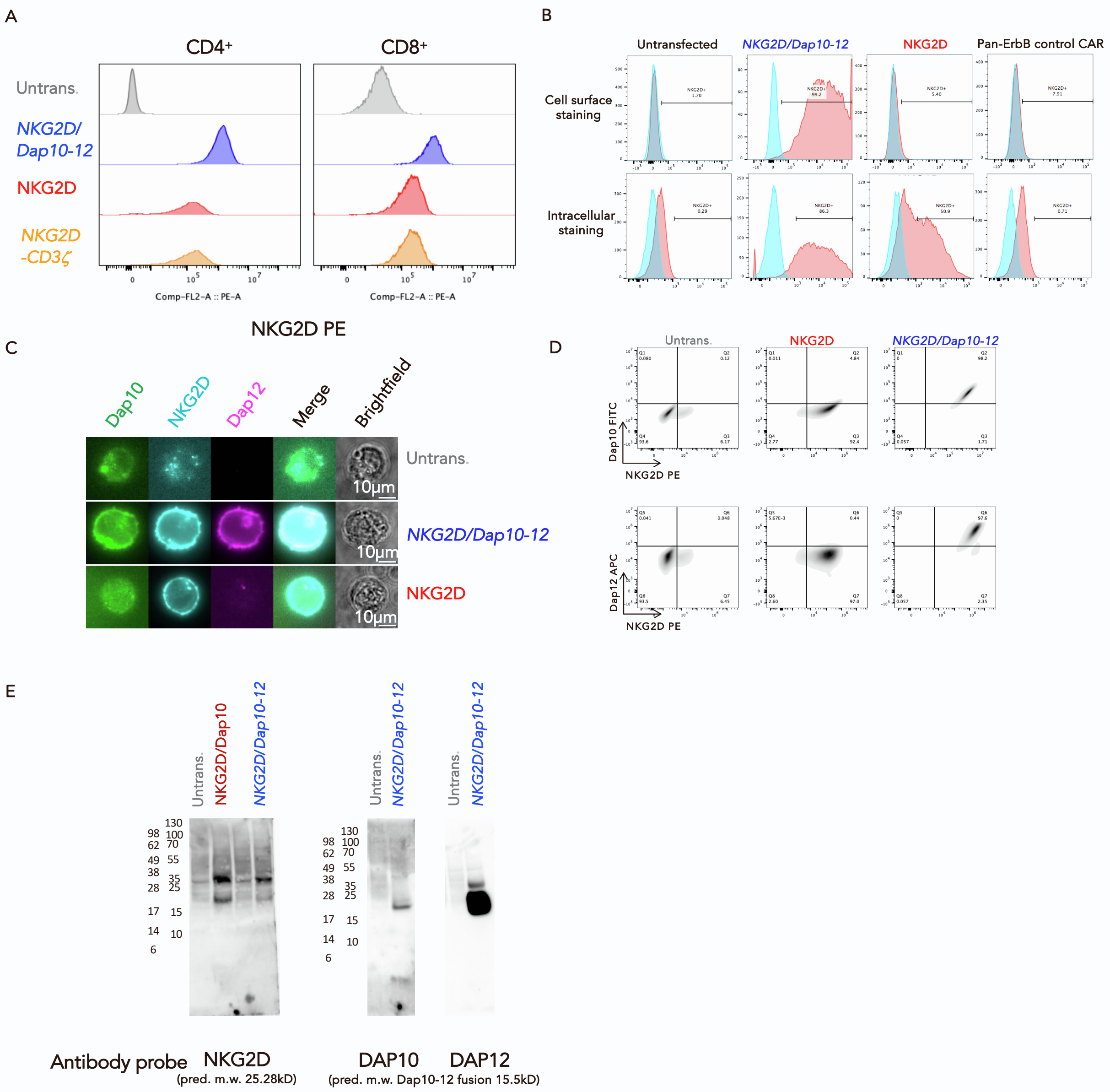

Figure S1. Confirmation of complex formation by NKG2D and Dap10-12 in *NKG2D/Dap10-12* T-cells

(A) Representative flow cytometric analysis of $CD4^+$ and $CD8^+$ T-cells engineered to express the *NKG2D/Dap10-12* CAR or *NKG2D-CD3ζ* CAR. T-cells in which NKG2D alone was over-expressed by retroviral transduction are shown as controls, together with untransduced (untrans.) T-cells. Data are representative of at least 3 independent replicates that gave similar results. (B) 293T cells were transfected with the indicated plasmids. NKG2D expression was detected by flow cytometry in non-permeabilized (cell surface staining) and permeabilized (intracellular staining) cells. A panErbB CAR plasmid (SFG T4) was used as an additional negative control. Data are representative of 3 independent replicates. (C) TIRF microscopy images of the indicated T-cell populations following incubation with Alexa Fluor® 488 anti-human DAP10 antibody, Alexa Fluor® 647 anti-human DAP12 antibody and PE anti-human NKG2D antibodies. Data are representative of 3 independent donors. (D) Upper panels show flow cytometric detection of NKG2D and Dap10 co-expression on the cell surface of the indicated non-permeabilized T-cell populations. Lower panels show co-expression of NKG2D and Dap12 in permeabilized *NKG2D/Dap10-12* T-cells. Data are representative of 3 independent replicates that gave similar results. (E) Western blotting was performed on lysates prepared from 293T cells that were untransfected (untrans.) or transfected with NKG2D/ Dap10-12. NKG2D/Dap10 is a control in which NKG2D is co-expressed with Dap10 alone. Predicted molecular weights of NKG2D and Dap10-12 without post translational modification are provided together with two molecular weight ladders run along with each blot.

Related to Figure 1.

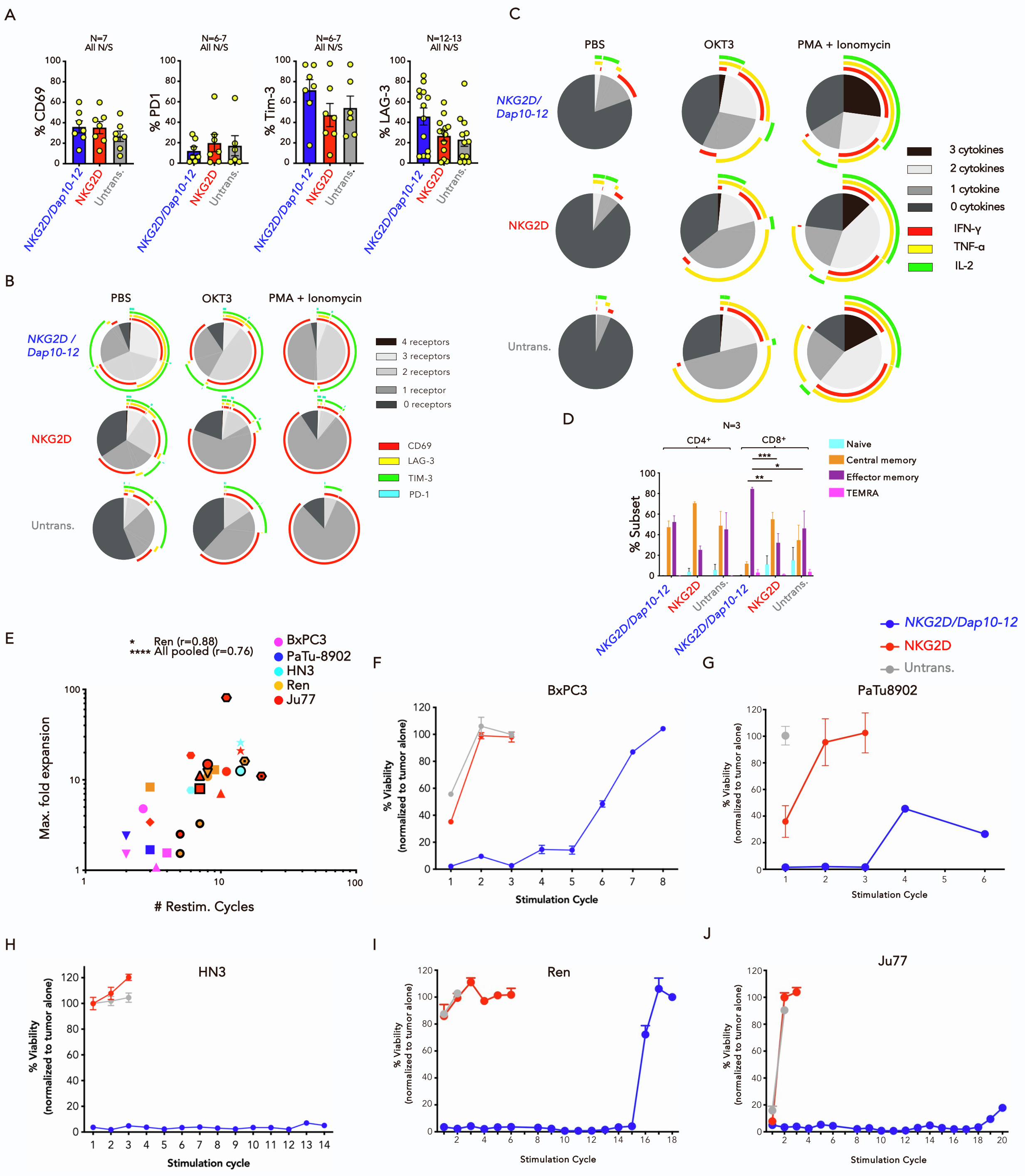

Figure S2. In vitro analysis of *NKG2D/Dap10-12* T-cells

(A) Flow cytometric analysis of CD69, PD1, Tim-3 and LAG-3 (all show mean ± SEM) expression on the indicated CAR and control T-cells. Number of independent biological replicates are indicated on this and subsequent panels as appropriate. N/S – not significant. (B) Spice analysis of activation/ exhaustion marker expression by T-cells engineered to express *NKG2D/Dap10-12*, NKG2D alone or untransduced as control. Plots are representative of two independent repeats. (C) *NKG2D/Dap10-12* CAR T-cells were stimulated for 24 hours as indicated, making comparison with the indicated control cells. Cytokine-producing cells were quantified using flow cytometry. Data are representative of three independent replicates. (D) Differentiation subset analysis of *NKG2D/Dap10-12* CAR T-cells on day 12 of culture, making comparison with the indicated control cells (mean ± SEM). $CD4^+$ and $CD8^+$ T-cell subsets were defined as naive ($CD45RO^-$ $CCR7^+$), central memory (CM; $CD45RO^+$ $CCR7^+$), effector memory (EM; $CD45RO^+$ $CCR7^-$) and terminally differentiated effector memory (TEMRA) cells ($CD45RO^-$ $CCR7^-$). *$p<0.05$; **$p<0.01$; ***$p<0.001$ by two-way ANOVA. (E) Correlation was assessed between number of productive re-stimulation cycles for individual donors (denoted by different symbols) and maximum fold expansion of *NKG2D/Dap10-12* CAR T-cells on the indicated tumor monolayers (denoted by indicated colors). *$p<0.05$; ****$p<0.0001$ by Spearman test for each individual tumor cell line and for a pooled analysis for all five tumor cell monolayers. Representative tumor re-stimulation assays performed on BxPC3 (F), PaTu8902 (G), HN3 (H), Ren (I) and Ju77 (J) tumor cell monolayers.

Related to Figure 1.

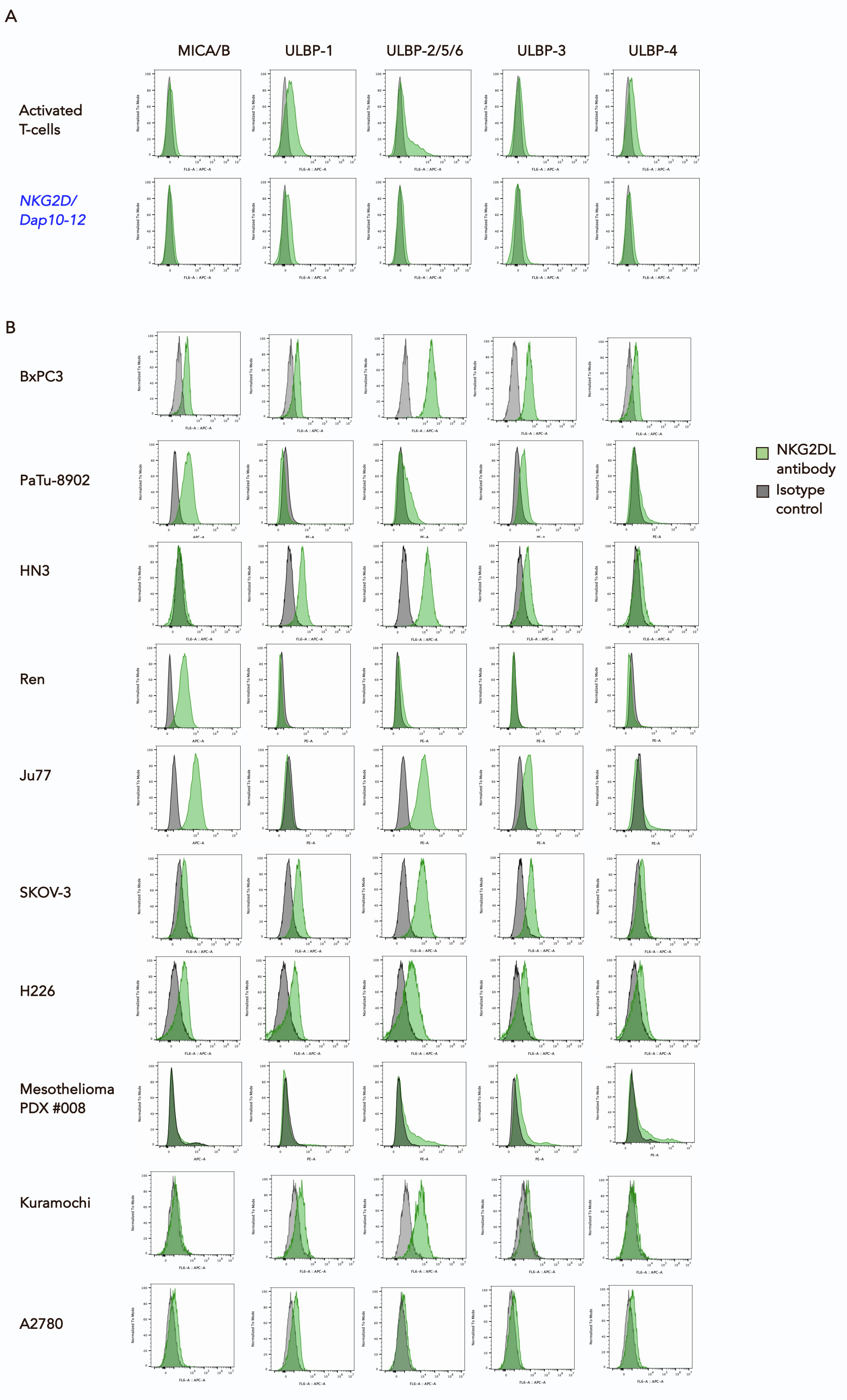

Figure S3. NKG2D ligand expression on tumor cell models and activated T-cells

Activated untransduced or *NKG2D/Dap10-12* CAR T-cells (A) and tumor cells used for in vitro and in vivo studies (B) were analyzed for expression of NKG2D ligands by flow cytometry. Data are representative of at least three independent replicates.

Related to Figure 1.

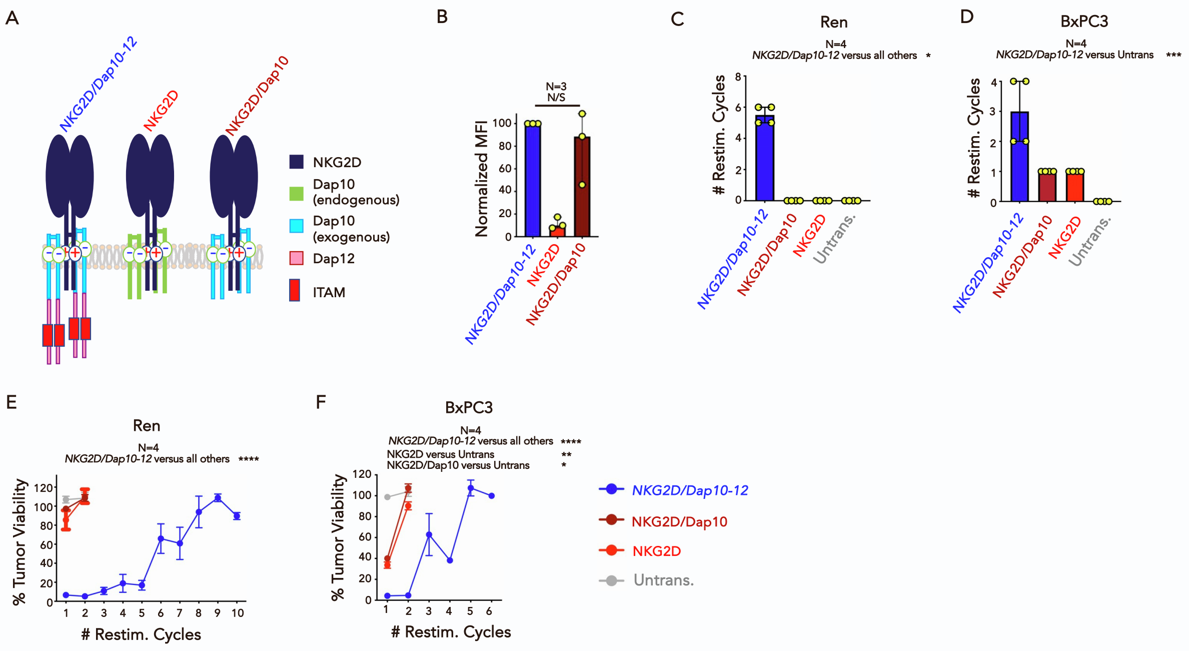

Figure S4. Comparison of *NKG2D/Dap10-12* with NKG2D/Dap10 control T-cells

(A) Function of *NKG2D/Dap10-12* CAR T-cells was compared to controls in which NKG2D alone or the combination of NKG2D and Dap10 were over-expressed. Note that NKG2D also associates with endogenous Dap10 in T-cells. (B) MFI of NKG2D expression in $CD4^+$ T-cells following transduction with the *NKG2D/Dap10-12* CAR, NKG2D alone or NKG2D/Dap10. Number of independent biological replicates is shown on this and subsequent panels as appropriate. Data were normalized to expression in *NKG2D/Dap10-12* CAR T-cells, which was set to 100. Error bars show median + interquartile range. Statistical analysis was by Kruskal-Wallis test; N/S – not significant. Number of effective re-stimulation (restim.) cycles achieved by *NKG2D/Dap10-12* and control T-cells when iteratively re-stimulated twice weekly on Ren (C) and BxPC3 (D) tumor cell lines. Re-stims were considered successful if <60% of tumor cells remained viable at the time of initiation of the next twice weekly stimulation cycle. Data show median + interquartile range. *p<0.05; ***p<0.001 by Kruskal Wallis test. Tumor viability was monitored after each restim cycle on Ren (E) and BxPC3 (F). Data show mean±SEM. *p<0.05; **p<0.01; ***p<0.001 by two-way ANOVA.

Related to Figure 1.

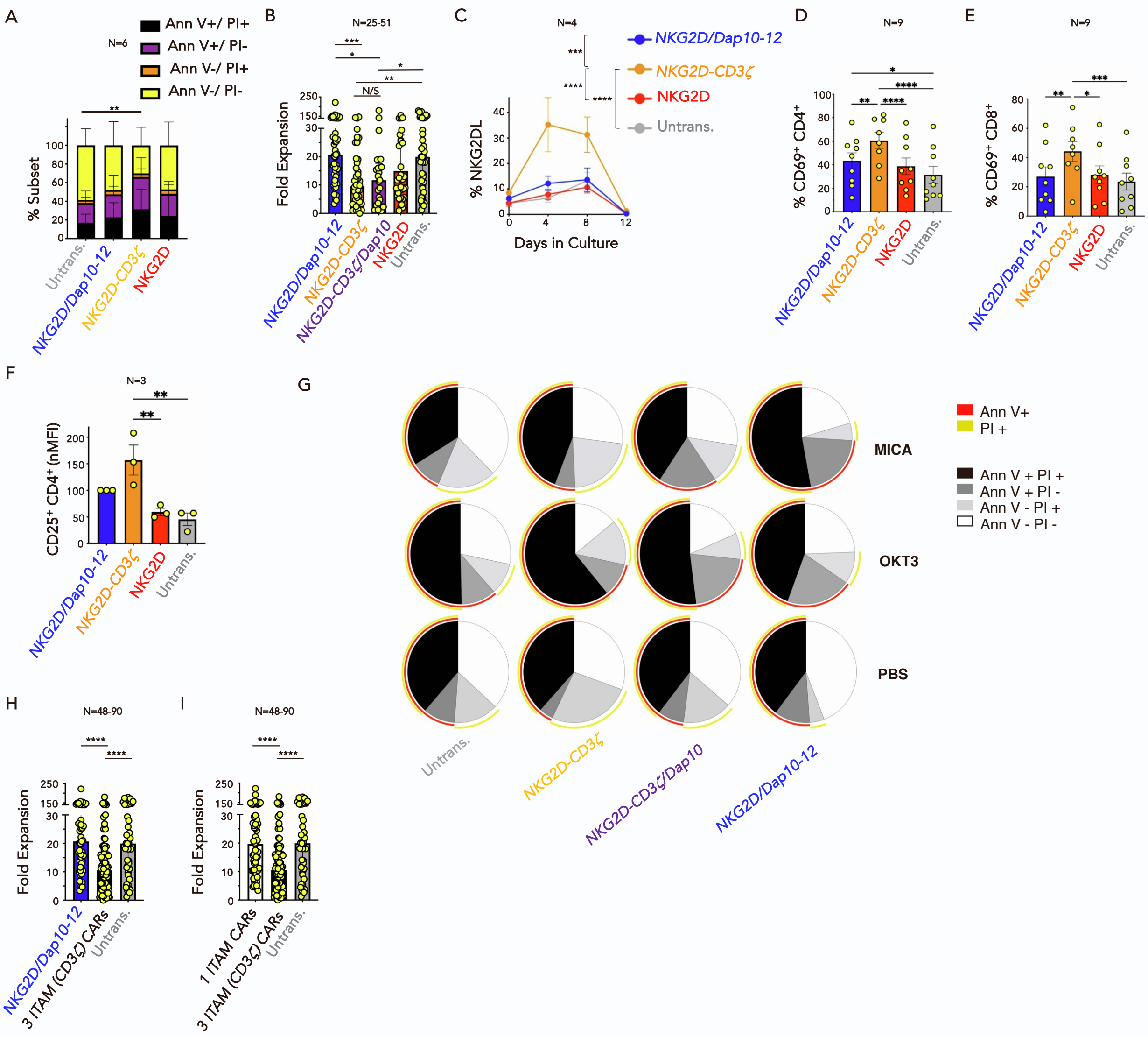

Figure S5. Analysis of viability, yield and activation state of the indicated CAR T-cell populations

(A) T-cells were engineered to express the indicated CARs or NKG2D alone and were analyzed for viability/ apoptosis by flow cytometry following staining with Annexin V (Ann V) and propidium iodide (PI). Mean +/- SD n=6 independent biological replicates. ***p*<0.01 using paired Student *t*-test for viable (i.e. Ann V-/ PI-) cells. (B) Expansion of the indicated CAR T-cell populations over 11 days post retroviral transduction. Number of independent biological replicates is shown on this and subsequent panels as appropriate. **p*<0.05, ***p*<0.01, ****p*<0.001 and N/S (not significant) by Kruskal-Wallis test. (C) Engineered T-cells were expanded in culture and analyzed for NKG2DL expression by flow cytometry using an NKG2D-Fc fusion protein to simultaneously detect all ligands (mean +̲ SEM; ****p*<0.001, *****p*<0.001 by two-way ANOVA. Following expansion in culture, the indicated CAR T-cells were analyzed by flow cytometry for CD69 ($CD4^+$ T-cells, D; $CD8^+$ T-cells, E) and CD25 ($CD4^+$ T-cells, F). In the case of CD25, since all cells were positive, MFI was normalized against that of *NKG2D/Dap10-12* which was set to 100 arbitrary units. **p*<0.05, ***p*<0.01, ****p*<0.001 and *****p*<0.0001 by one-way ANOVA. (G) T-cells that expressed the indicated CARs or NKG2D as control were stimulated for 24 hours on immobilized MICA, OKT3 or with PBS as control. To quantify activation-induced cell death, cells were analyzed for viability/ apoptosis by flow cytometry following staining with Ann V and PI. (H) Expansion of the indicated CAR T-cell populations over 11 days post retroviral transduction. The 3 ITAM (CD3ζ) CARs group comprised *NKG2D-CD3ζ*, *NKG2D-CD3ζ/Dap10* and *NKG2D/Dap10-CD3ζ*. *****p*<0.0001 by Kruskal-Wallis test. (I) Expansion of the indicated CAR T-cell populations over 11 days post retroviral transduction. The 1 ITAM CARs group comprised *NKG2D/Dap10-12*, *NKG2D-Dap10-12*, *N(Tr.)-Dap10-12*, *NKG2D-Dap10-12/Dap10*, *N(Tr.)-Dap10-12/Dap10* and *NKG2D/Dap10-CD3ζ(1XX)*. The 3 ITAM (CD3ζ) CARs group comprised *NKG2D-CD3ζ*, *NKG2D-CD3ζ/Dap10* and *NKG2D/Dap10-CD3ζ*. *****p*<0.0001 by Kruskal-Wallis test.

Related to Figure 3.

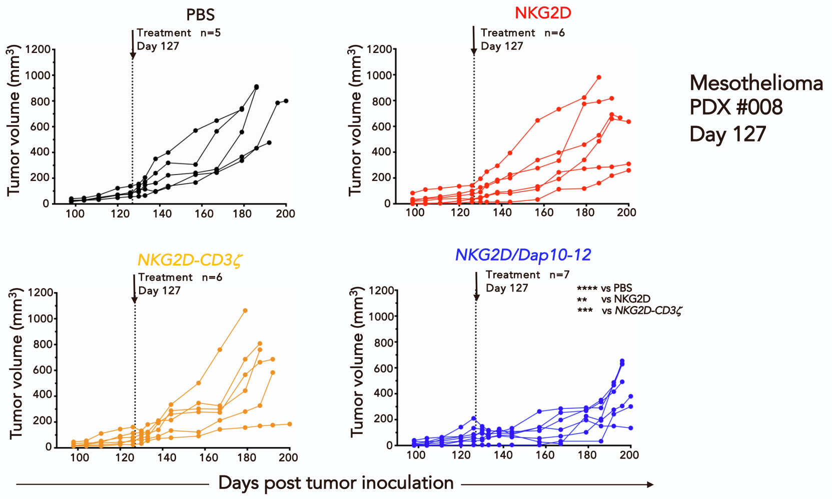

Figure S6. In vivo comparison of *NKG2D/Dap10-12* and *NKG2D-CD3ζ* CAR T-cells against advanced mesothelioma PDX tumors

Small chunks (approximately 2mm x 2mm) of mesothelioma PDX_008 were injected subcutaneously in NSG mice. Once tumors had established for 127 days, 4 million of the indicated CAR T-cell populations were injected i.v., making comparison with PBS and T-cells in which NKG2D alone was over-expressed. Number of independent biological replicates is indicated. Tumor volume was monited by caliper measurements. $^{**}p<0.01$; $^{***}p<0.001$; $^{****}p<0.0001$ using two-way ANOVA.

Related to Figure 4.

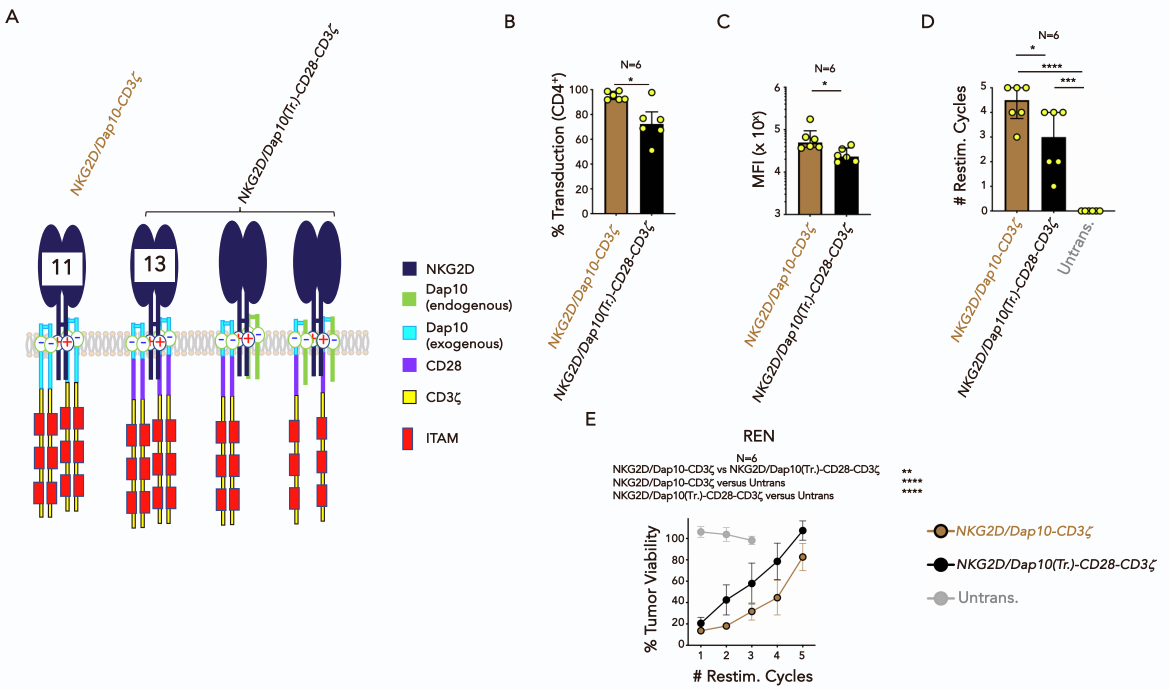

Figure S7. Evaluation of CD28-containing NKG2D-based CAR designs

(A) Function of CD28 versus Dap10 was compared using the indicated CAR designs. Numbering shown is to match these CARs with those shown in Figure 5. Additional predicted structures shown for *NKG2D/Dap10(Tr.)-CD28-CD3ζ* reflect the ability of NKG2D to also associated with endogenous Dap10 present in T-cells. (B) Transduction efficiency of the indicated CARs was assessed by flow cytometry, measuring percentage cell surface NKG2D in the $CD4^+$ T-cell subset. Number of independent biological replicates is shown on this and subsequent panels. Error bars show median + interquartile range. *$p<0.05$ by Mann Whitney test. (C) Mean fluorescence intensity of NKG2D expression in $CD4^+$ T-cells following transduction with the indicated CARs. Error bars show median + interquartile range. *$p<0.05$ by Mann Whitney test. (D) Number of effective re-stim cycles achieved by the indicated CAR T-cells when iteratively re-stimulated twice weekly on Ren tumor cells. Re-stims were considered successful if <60% of tumor cells remained viable. Data show median + interquartile range. *$p<0.05$; ***$p<0.001$; ****$p<0.0001$ by one-way ANOVA. (E) Tumor viability was monitored after each restim cycle on Ren cells. Data show mean$\pm$SEM. **$p<0.01$; ****$p<0.0001$ by two-way ANOVA.

Related to Figure 5.
